# Tumor-derived systems as novel biomedical tools—turning the enemy into an ally

**DOI:** 10.1186/s40824-023-00445-z

**Published:** 2023-11-09

**Authors:** Nimeet Desai, Pratik Katare, Vaishali Makwana, Sagar Salave, Lalitkumar K. Vora, Jyotsnendu Giri

**Affiliations:** 1 Department of Biomedical Engineering, Indian Institute of Technology Hyderabad, Kandi, Telangana India; 2 Center for Interdisciplinary Programs, Indian Institute of Technology Hyderabad, Kandi, Telangana India; 3 Department of Pharmaceutics, National Institute of Pharmaceutical Education and Research-Ahmedabad (NIPER-A), Gujarat, India; 4 School of Pharmacy, Queen’s University Belfast, 97 Lisburn Road, Belfast, BT9 7BL UK

**Keywords:** Cancer cell membrane, Extracellular vesicles, Tumor cell lysate, Tumor organoids, Cancer, Cancer research

## Abstract

Cancer is a complex illness that presents significant challenges in its understanding and treatment. The classic definition, "a group of diseases characterized by the uncontrolled growth and spread of abnormal cells in the body," fails to convey the intricate interaction between the many entities involved in cancer. Recent advancements in the field of cancer research have shed light on the role played by individual cancer cells and the tumor microenvironment as a whole in tumor development and progression. This breakthrough enables the utilization of the tumor and its components as biological tools, opening new possibilities. This article delves deeply into the concept of "tumor-derived systems”, an umbrella term for tools sourced from the tumor that aid in combatting it. It includes cancer cell membrane-coated nanoparticles (for tumor theranostics), extracellular vesicles (for tumor diagnosis/therapy), tumor cell lysates (for cancer vaccine development), and engineered cancer cells/organoids (for cancer research). This review seeks to offer a complete overview of the tumor-derived materials that are utilized in cancer research, as well as their current stages of development and implementation. It is aimed primarily at researchers working at the interface of cancer biology and biomedical engineering, and it provides vital insights into this fast-growing topic.

**Graphical Abstract:**

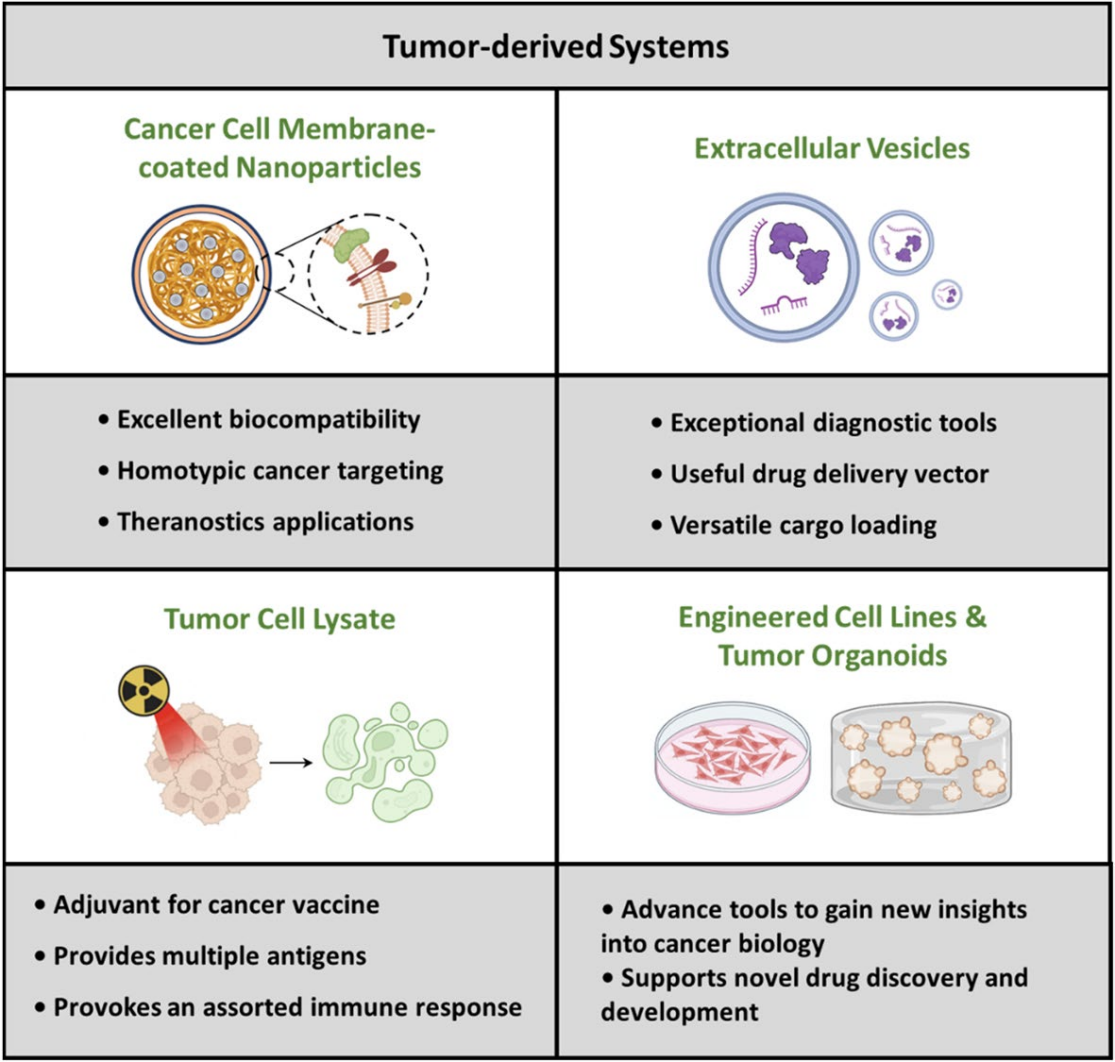

## Introduction

### Global cancer burden and treatment strategies

Cancer represents a heterogeneous group of diseases characterized by the uncontrolled proliferation of abnormal cells within the human body. Cancerous cells do not adhere to the programmed growth, division, and apoptosis cycle, unlike healthy cells. Their origins are diverse, stemming from various cell types, and the causative factors are typically multifaceted, including genetic mutations, exposure to carcinogenic agents, hereditary predisposition, and the aging process [1]. These rogue cells are notorious for subverting the body's intrinsic mechanisms that regulate cell growth and repair, ultimately leading to the formation of tumors. Furthermore, they can disseminate to distant anatomical sites via either the bloodstream or the lymphatic system, a phenomenon recognized as metastasis. This invasive potential constitutes a cardinal hallmark of cancer and is closely associated with an unfavorable prognosis [2]. Cancer has emerged as a global health crisis, substantially impacting public well-being. A report published by the World Health Organization in 2019 revealed that cancer accounted for three out of every ten premature deaths attributable to noncommunicable diseases in 183 countries [3]. 2020 witnessed a significant global burden of cancer, with a staggering 19.3 million new cases diagnosed and 10 million cancer-related fatalities recorded. Unfortunately, this concerning trend is expected to persist, with projections indicating a substantial surge in cancer incidence in the coming years. By 2040, it is estimated that an alarming 28.4 million new cases of cancer will be reported worldwide [4]. The escalating incidence of cancer underscores the urgent need to develop effective strategies to comprehensively understand and combat this malignancy.

Significant strides have been made in cancer research over recent decades. Researchers across the globe have been diligently dedicated to unraveling the intricate facets of this disorder, and as our comprehension deepens, therapeutic interventions consistently yield promising clinical outcomes. For decades, the pillars of cancer treatment have been surgical procedures, radiation therapy, and chemotherapy, with their utilization becoming increasingly refined through advancements in technology and expanding knowledge. The progression of surgical techniques and the incorporation of robotic systems have empowered surgeons to execute intricate procedures with heightened precision, minimal invasiveness, and reduced recovery periods [5]. Radiation therapy has also undergone a transformative journey, transitioning from conventional external beam radiation to cutting-edge methodologies such as proton therapy, stereotactic body radiation therapy, and intensity-modulated radiation therapy, enhancing the precision of cancerous tissue targeting while minimizing exposure to healthy tissue [6]. Similarly, innovative chemotherapeutic agents have been meticulously engineered to selectively target distinct molecular pathways and cellular mechanisms implicated in cancer proliferation and survival, rendering them more efficacious and less deleterious than traditional chemotherapy agents [7]. In conjunction with conventional therapies, these pioneering treatment modalities have notably elevated overall survival rates across various cancer types and have opened avenues for managing malignancies that were once deemed intractable.

Progress in diagnostic interventions has significantly contributed to enhancing the depth of insight into a patient's cancer condition. The utilization of advanced imaging techniques, such as positron emission tomography, computed tomography, and magnetic resonance imaging (MRI), has revolutionized our ability to access real-time visual data about a tumor's location, dimensions, and staging [8]. Concurrently, biopsies have evolved to furnish exceedingly precise and specific information concerning the molecular attributes of tumors and the presence of genetic mutations or variations [9]. These strides in diagnostic interventions have substantially elevated the precision of cancer diagnosis and facilitated tailored treatment strategies, yielding superior patient outcomes.

### Overview of tumor-derived systems

An exciting and evolving field within cancer research involves the development of biomaterial-based platforms tailored for precise and localized delivery of anticancer drugs, immunomodulatory biomolecules and diagnostic contrast agents. In most cases, these platforms integrate targeting ligands, facilitating precise interactions with diverse cellular elements within the intricate context of the tumor microenvironment (TME) [10]. Predominantly, these constituents encompass cancer cells, which can be selectively targeted through ligands such as arginine-glycine-aspartic acid peptide [11], folate [12], transferrin [13], or hyaluronic acid [14]. Furthermore, the targeting approach can also be extended to immune cells to directly modulate their anticancer immune responses. Dendritic cells (DCs), for instance, can be effectively modulated by exploiting surface receptors such as mannose receptors [15], Fc receptors [16], and Toll-like receptors [17] to enhance their functional capabilities. Reprogramming tumor-associated macrophages (TAMs) can be accomplished through selective targeting strategies, leveraging the overexpression of IL-4 and galactose-type lectin receptors [18, 19]. In parallel, the immunosuppressive functions of myeloid-derived suppressor cells (MDSCs) can be attenuated by targeting specific myeloid cell markers, such as CD11b^+^ and CD33^+^ [20]. Finally, nonimmune cells such as cancer-associated fibroblasts (CAFs), which play a pivotal role in fostering tumor growth by facilitating the remodeling of the cancer extracellular matrix (ECM), can be effectively targeted through surface markers such as fibroblast activation proteins [21].

While this approach offers flexibility in selecting the optimal delivery site and has shown promising results, as evident from the extensive academic literature on the subject, its clinical translation remains limited [22]. The primary obstacle lies in the complexity of the manufacturing process, which hampers scalability and results in elevated production costs, rendering the final product inaccessible to end users [23]. Consequently, despite the availability of numerous naturally sourced and synthetic biomaterials, the scientific community continues to explore and develop superior alternatives.

Profound insights and practical solutions are often achieved through a deep and comprehensive understanding of the problem at hand. This principle holds true in various aspects of life, including the realm of biomedical science. Taking this into account, certain cellular characteristics of tumors can be exploited for biomedical purposes. Notably, tumor-derived extracellular vesicles (EVs) play a pivotal role in mediating the exchange of molecular components and signaling events, collectively contributing to the progression of pathological conditions. These EVs possess remarkable attributes, such as high stability, versatility in cargo loading, and excellent biocompatibility, rendering them valuable candidates for drug delivery systems [24]. When sourced from tumors, EVs bear specific surface markers that function as intrinsic ligands, facilitating targeted interactions with cancer cells and specific immune cells within the TME. Furthermore, the profiling of exosomal cargo, encompassing nucleic acids, proteins, and lipids, is a vital diagnostic tool for assessing and monitoring tumor progression [25].

In addition to EVs, the cancer cell membrane (CCM) plays a critical role in tumor development, progression, and metastasis. Altered membrane composition, structure, and functionality equip cancer cells with the ability to thrive in hostile environments, evade immune surveillance, and migrate to distant locations. When isolated in a functionally viable state, CCM can be harnessed as a fundamental component in the emerging class of nanocarriers termed CCM-coated nanoparticles. Comprising a synthetic nanoparticulate core loaded with drugs or contrast agents concealed by a layer of cancer cell-derived membrane, this approach imparts a "biomimetic" character to conventional nanoparticles. This strategy offers enhanced biocompatibility, immune evasion, and homotypic tumor targeting [26, 27].

Beyond the therapeutic and diagnostic potential of EVs and CCM, tumors themselves can be engineered as a modality for comprehending and combating cancer. The induction of an effective and specific immune response against cancer cells is pivotal to successful immunotherapy. While multiple antigens can be targeted for this purpose, the utilization of immunogenically dying tumor cells or tumor cell lysate (TCL) offers a comprehensive array of epitopes, promoting a multivalent immune response [28]. Finally, cancerous tissues can be cultured to establish cancer cell lines, which are the foundational cornerstone of cancer research. Progress in biotechnology and molecular science has empowered the genetic manipulation of these cancer cell lines, further expanding their utility in cancer research. Notably, they play a pivotal role in unraveling disease biology and advancing the development of novel biomolecular interventions [29].

**Fig. 1:**
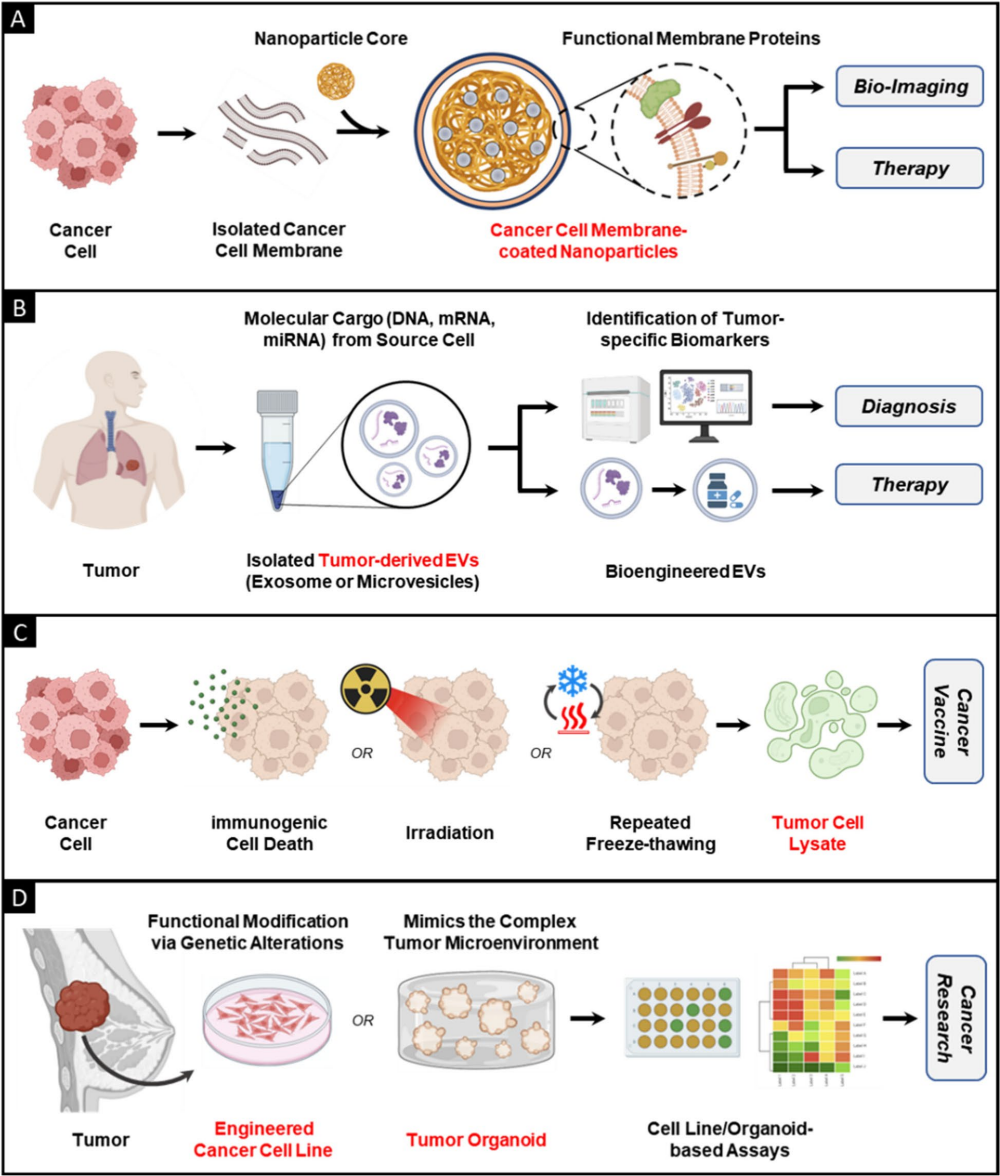
Schematic representing different categories of tumor-derived systems and their utilization as biomedical tools. **A** Potentiating tumor targeting by coating nanoparticles with functional CCM. **B** Employing tumor-derived EVs as diagnostic tools and delivery vectors for anticancer therapy. **C** Developing TCL as a potent cancer vaccine component. **D** Employing cancer cell lines and tumor organoids as multipurpose tools in cancer research

The aforementioned systems can be collectively grouped as “tumor-derived biomedical tools,” as illustrated in Fig. 1. These systems possess a multitude of distinctive attributes that have attracted substantial attention in the field of bioengineering for potential clinical applications.

### About this manuscript

This comprehensive review aims to thoroughly analyze tumor-derived systems, highlighting their state-of-the-art applications in various areas, such as drug delivery, immunotherapy, cancer detection and diagnosis, vaccine development, and fundamental cancer research.

Through an extensive survey of the latest literature, several outstanding research studies showcasing these systems' immense potential in the fight against cancer were identified. The reference selection process was conducted systematically to ensure the sources' highest quality and relevance. Specific criteria for inclusion were established, encompassing factors such as relevance to the topic, recency of publication, credibility of the source, and the significance of the study. To retrieve the relevant literature, comprehensive keyword searches were conducted on PubMed. These searches were performed from 2003 to 2023, and the search strategy included using these keywords and filters as per PubMed's capabilities.

**Fig. 2:**
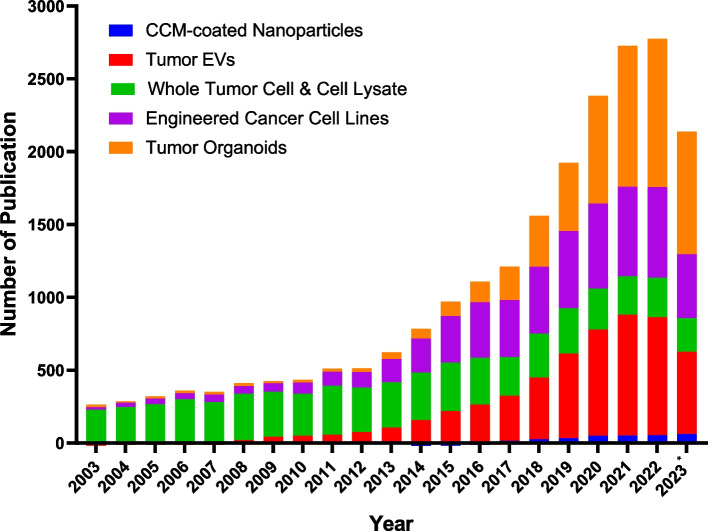
Graphical plot illustrating the number of scientific papers published over the last two decades (from 2003 to 21 September 2023) on biomedical systems that can be categorized as “tumor-derived.” These papers were systematically identified in the Pubmed database by employing keywords and filters tailored to this research focus

Subsequently, the search results were meticulously reviewed to ensure the utmost rigor in their use. The retrieved reference was subjected to a comprehensive evaluation, carefully considering how well they aligned with predefined criteria for inclusion. The grounds on which some studies were excluded included factors such as a lack of direct relevance to the subject matter, outdated information, unreliable/questionable sources, or studies that did not contribute substantively to the overarching theme of this review. Figure 2 depicts the yearwise distribution of publications (from 2003 to 2023) sourced from our comprehensive searches on PubMed. It provides a visual depiction of the evolving research landscape in the field over the past several years.

The subsequent sections of this review paper are structured to address distinct categories of tumor-derived systems. Drawing from an extensive review of the literature, comprehensive insights encompassing fundamental principles, the developmental or fabrication process, and a case-based examination of their practical utility have been presented. It is pertinent to mention that, for brevity and reader engagement, our discussions pertaining to the application aspects are primarily based on select studies that furnish distinctive insights, effectively showcasing the versatile utility of these tumor-derived systems. This strategic approach is implemented to enhance the readability and overall enjoyment of the manuscript for our readers while maintaining scientific rigor.

## Cancer cell membrane

### Understanding CCM

In recent years, the field of cancer treatment has undergone a revolutionary transformation with the introduction of novel anticancer drugs as integral components of chemotherapy regimens. The lack of selectivity, systemic cytotoxicity, and occurrence of multidrug resistance have provided a significant obstacle to effectively utilizing these anticancer drugs [30]. In this context, strategies that facilitate tumor-targeted delivery by encapsulating these drugs into nanoparticles have demonstrated excellent promise in improving treatment effectiveness. The recent rise of nanoparticle-derived products receiving regulatory approval is a testament to their clinical feasibility [31]. However, it is worth mentioning that the majority of these marketed nanoparticles rely solely on the “enhanced permeability and retention” (EPR) effect (a passive targeting approach that takes advantage of the tumor’s leaky vessels and the poor lymphatic system) to reach the tumor vicinity [32]. In the absence of an active targeting approach, the exceedingly heterogeneous nature of vessel fenestrations (for most types of tumors) or the lack of leaky vasculature (as observed in slow-growing tumors) acts as a biological barrier that restricts the overall reach of nanoparticles, indirectly requiring a higher concentration of nanoparticles to be administered [33]. Furthermore, these nanoparticles may face immune recognition challenges contingent upon their particle dimensions and chemical composition. This recognition can result in their premature elimination from the bloodstream, preventing them from reaching the targeted tumor site [34]. Biomimetic functionalization, especially using CCM, has evolved as a lucrative approach to endow nanoparticles with more prolonged circulation, effective delivery, and active targeting [35].

The use of CCM endows nanoparticles with several distinct advantages. First, if isolated from source cancer cells or patient‐derived xenografts, CCM provides a unique opportunity to design personalized treatment for addressing cancer heterogeneity due to the coexistence of several cell types with varied phenotypes. Improved patient specificity yields better clinical outcomes [36]. Second, the endogenous nature of CCM confers superior biocompatibility and safety. Finally, particles coated/camouflaged with CCM demonstrate extended systemic circulation without eliciting an immune response, facilitating remarkable tumor site-specific localization. This feat is achieved without the need to employ complex surface modifications, as seen in other active targeting strategies such as biological ligands, targeting peptides, or aptamers [37, 38]. Cancer cell adhesion molecules (CCAMs) are the common underlying reason for the abovementioned advantages associated with CCM. CCAMs encompass various membrane receptors, chiefly including (but not limited to) the immunoglobulin superfamily (Ig-SF), selectins, integrins, and cadherins [39–41]. CCAMs are pivotal in establishing cell‒cell and cell-ECM interactions and are indirectly responsible for cancer recurrence, invasion, and metastasis [42]. From a functional perspective, cadherins (mainly cadherin-1, 2, and 19; protocadherin) aid in cell‒cell adhesion within tumors by regulating cell migration and gene regulation (through catenin pathways) [43]. Integrins (mainly IT-β1, β3, β4, and β5; IT-α1, α2, and α5) are essential for cell proliferation, differentiation, and migration due to their capacity to transmit ECM signals to the cell [44]. Selectins and Ig-SF members (ALCAM, Contactin, ICAM, MCAM, NCAM) activate signaling cascades that promote malignant behavior by negatively modulating the immune cells within the TME [45, 46]. In addition to CCAMs, the surface of a cancer cell is also enriched with CD47. This ubiquitous transmembrane protein interacts predominantly with signal regulatory protein-α expressed by macrophages and DCs [47]. It primarily functions as a "do not eat me" signal by activating protein phosphatases and inhibiting immune phagocytosis [48]. By applying suitable CCM isolation techniques, these membrane receptors can be retained in a functional state, and their subsequent coating onto nanoparticles allows them to engage in homotypic adhesive interactions (with tumors) and evade systemic immune cells [49].

### Leveraging CCM for biomimetic functionalization

Fang et al*.* [50] were among the first groups to study the homologous binding mechanisms of nanoparticles coated with CCM. Compared to bare nanoparticles or nanoparticles coated with erythrocyte membrane, the authors found that poly(lactic-co-glycolic acid) (PLGA) nanoparticles coated with cell membrane isolated from the MDA-MB-435 metastatic cancer cell line underwent substantial cellular attachment to the source cells. The cancer cell-specific affinity of the CCM coating was further validated when the CCM-coated PLGA core displayed a similar uptake profile to the bare PLGA core when incubated with human foreskin fibroblasts (as a negative control). After this pioneering study, CCM isolated from various cell lines has been widely reported [51]. Regardless of the cancer cell type, CCM can be isolated using a simple and scalable top-down approach. The standard procedure for membrane isolation typically entails treating the source cells with a hypotonic lysis buffer comprising various components, including phenylmethyl sulfonyl fluoride, sodium bicarbonate, and ethylenediaminetetraacetic acid. Following incubation in lysis buffer, the cells were disrupted by gentle homogenization using mechanical pressure (preferably with a Dounce homogenizer). Recently, many commercial membrane protein extraction kits have become available on the market that contain proprietary buffer solutions that replace homogenization with sequential freeze‒thaw cycles (by submerging the cells into liquid nitrogen) [52]. In both scenarios, the cell lysate obtained is subjected to thorough differential ultracentrifugation, leading to the isolation of a purified CCM pellet. The final CCM vesicles are typically prepared by washing them once with a solution containing Tris-hydrochloride and ethylenediaminetetraacetic acid, followed by physical extrusion through a 400-nm polycarbonate (PC) membrane [53]. Any nanoparticle can be coated with these isolated CCM vesicles by coextrusion through a PC membrane of lower pore size [54]. Other reported methods to coat CCM include sonication (after prior incubation) and microfluidic-assisted nanoparticle coating. The presence of functional membrane surface receptors can be validated using western blotting, whereas the effective coating of CCM on the nanoparticle can be assessed using transmission electron microscopy (TEM) [55]. Measurement of the hydrodynamic diameter and zeta potential using photon correlation spectroscopy (also known as dynamic light scattering) can provide semiquantitative confirmation of nanoparticle coating [56].

**Fig. 3:**
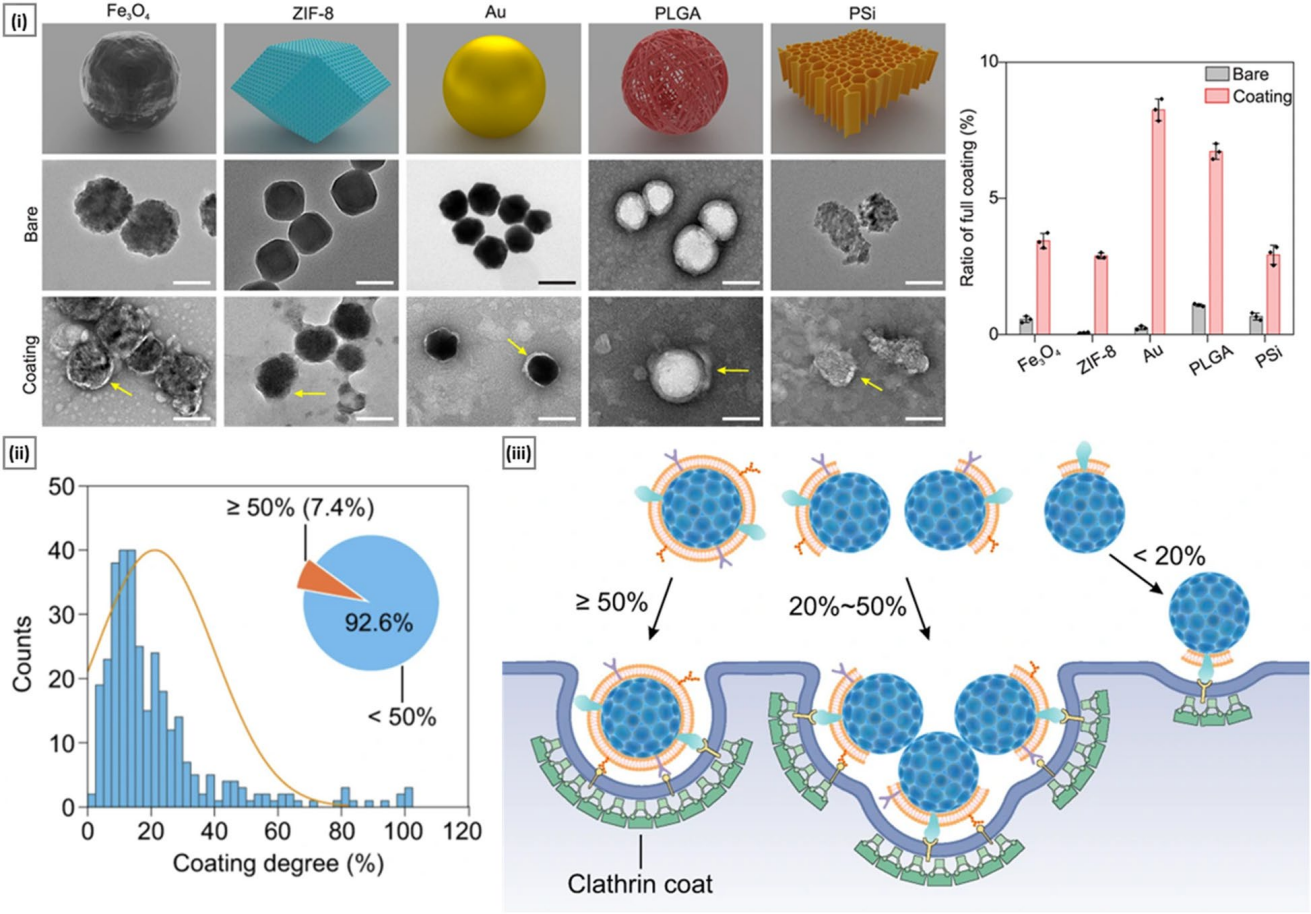
Assessment of cell membrane coating integrity and its impact on the internalization mechanism. In subfigure (i), TEM images are presented for various nanoparticle cores (Fe_3_O_4_, ZIF-8, Au, PLGA, and porous silicon) before and after cell membrane coating, accompanied by quantification of the ratio of complete cell membrane coating (Scale bar: 100 nm). Subfigure (ii) displays the distribution of cell membrane coating degrees on a SiO_2_ core, determined from TEM images (*n* = 325). The inset provides information on the proportion of SiO_2_ cores with a low cell membrane coating degree, specifically below 50%. In subfigure (iii), a schematic illustration is presented, elucidating potential endocytic entry mechanisms for partially coated cores. Adapted with permission from [57] (Copyright Springer Nature, 2021)

While the premise of CCM-coated nanoparticles is lucrative, it should be noted that the current gold-standard protocols to coat nanoparticles with CCM are not fully efficient. In a recent study, Liu et al*.* [57] introduced a fluorescence quenching assay to assess the integrity of the cell membrane coating. By studying the coating of CMM (isolated from diverse cell types such as CT26 cells and HeLa cells) with multiple nanoparticulate cores (magnetite, gold, PLGA, and porous silicon), the authors demonstrated that up to 90% of the nanoparticles are only partially coated (with more than 60% of the sample population having a coating degree < 20%). In in vitro homologous targeting studies, it was observed that the nanoparticles, despite being partially coated, were still capable of being internalized by the target cells. Extensive molecular simulations were conducted to gain further insights into the endocytic entry mechanism. Based on these simulations, the authors proposed that nanoparticles with a high coating degree (≥ 50%) enter the cells individually, whereas those with a low coating degree (< 50%) require aggregation before internalization (Fig. 3). In a follow-up study, the authors explored the addition of external phospholipids as “helpers” to enhance CCM fluidity and promote the final fusion of lipid patches. The nanoparticles coated with this method showed a high ratio of complete coating (23%) and superior tumor-targeting capabilities compared to nanoparticles coated conventionally [58]. In addition to external phospholipids, designing “hybrid membranes” by combining CCM with membrane vesicles isolated from other cell sources, such as erythrocytes, platelets, immune cells (macrophages, cytotoxic T lymphocytes (CTLs)), and cellular TME components (MDSCs, CAFs), has been explored [59, 60].

### Applications in tumor theranostics

The inherent ability of CCM-coated nanoparticles to localize in close proximity to the TME has been leveraged to design several advanced cancer theranostics platforms [61]. This approach benefits from diverse core materials, ranging from natural biomaterials such as chitosan, alginate, and silk fibroin to synthetic systems such as polymeric, lipid-based, or metallic nanosystems [62, 63]. Depending on the intended application, the core can be equipped with functional cargo such as biomolecules, immune adjuvants, or contrast agents [64]. Some recent applications are discussed below.

**Fig. 4:**
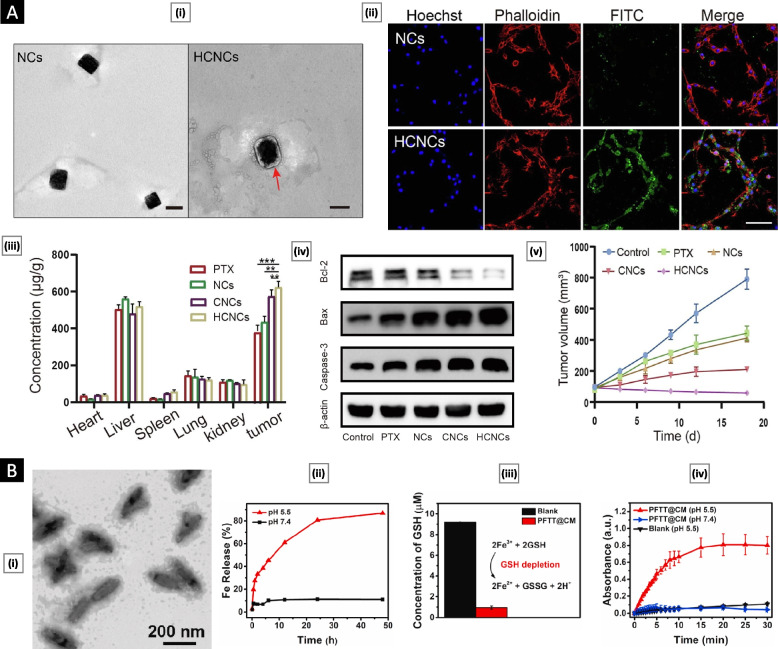
**A** Herceptin-functionalized CCM-coated PTX nanocrystals for targeted therapy of HER2-positive breast cancer. Subfigure (i) displays TEM images comparing uncoated NCs and HCNCs, revealing successful coating (scale bar: 200 nm). Subfigure (ii) exhibits CLSM images illustrating the cellular uptake of FITC-labeled NCs and HCNCs in SK-BR-3 cells, demonstrating enhanced uptake by HCNCs (Scale bars: 50 µm). Subfigure (iii) demonstrates the biodistribution patterns of HCNCs in tumors and main organs. Figure (iv) shows the western blot analysis of β-actin, caspase-3, Bax, and Bcl-2 proteins, enabling the assessment of the apoptotic capability of HCNCs. Finally, subfigure (v) depicts the in vivo antitumor effect of HCNCs. Adapted with permission from [65] Copyright Elsevier, 2022). **B** CCM-coated MOF for multimodal tumor therapy. Subfigure (i) provides TEM images of PFTT@CM, showing the structure of the MOF coated with CCM. Subfigure (ii) demonstrates the cumulative release of Fe3+ from PFTT@CM at different pH levels, indicating its acid-dependent dissociation. Subfigure (iii) presents the ability of PFTT@CM, mediated by Fe.3+, to deplete GSH. Subfigure (iv) reveals the continuous catalysis of H2O2 by PFTT@CM, monitored through a 3,3′,5,5′-tetramethylbenzidine assay over a 30-min duration. Adapted with permission from [66], (Copyright Elsevier, 2022)

To tackle critical challenges in cancer treatment, such as low drug loading, poor solubility of anticancer drugs, and targeting specificity, Wu et al*.* [65] devised a sophisticated system comprising paclitaxel (PTX) nanocrystals coated with the SK-BR-3 cell membrane. The coated membrane was modified with Herceptin (a monoclonal antibody that selectively binds to HER2 receptors), making the final platform (HCNCs) an excellent candidate for treating HER2-positive breast cancer. Upon analysis via TEM, HCNCs displayed a characteristic cubic shape and measured approximately 220 nm in size. The authors utilized FITC-labeled HCNCs to investigate cellular uptake and homotypic targeting. Findings from confocal laser scanning microscopy (CLSM) indicated significantly elevated uptake of HCNCs compared to uncoated nanocrystals. Notably, this enhanced uptake was predominantly observed in SK-BR-3 cells, underscoring the platform's selectivity. In a BALB/c nude mouse model, the intravenous administration of HCNCs demonstrated remarkable tumor localization with minimal unintended distribution to vital organs. The inclusion of Herceptin further potentiated the platform's therapeutic effects. HCNCs exhibited a proapoptotic capability, as evidenced by the upregulation of proapoptotic proteins, including caspase-3 and Bax, alongside a corresponding decrease in the antiapoptotic protein Bcl-2. HCNCs achieved an in vivo tumor inhibition rate of 83.1 ± 3.54% (Fig. 4A). The positive result highlights CCM’s ability to potentiate conventional nanosystems by imparting a selective targeting ability.

To enhance cancer immunotherapy, Li et al*.* [67] developed a biomimetic system (termed CFIN) by combining triblock polymer-based nanomicelles with 4T1 CCM. The nanomicelles were loaded with indocyanine green (ICG, a photothermal agent) and NLG919 (an effective IDO-1 enzyme inhibitor). This system addresses the challenge of poor tumor immunogenicity by combining photothermal and immune therapies. CFIN are approximately 220 nm in size and have a negative surface charge of -23 mV. When exposed to an 808 nm laser for 10 min, they exhibited excellent photothermal properties, increasing their temperature by 34.5 °C. Under laser irradiation for just 1 min, CFIN effectively reduced cell viability to less than 4% in 4T1 cells. This result highlighted the ability of CFIN to ablate cancer cells through efficient photothermal energy conversion. Moreover, CFIN induced immunogenic cell death (ICD), promoting the expression of calreticulin and stimulating DC phagocytosis. In a murine 4T1 tumor model, CFIN treatment combined with laser irradiation led to nearly complete inhibition of primary tumor growth (93.5% inhibition rate) and delayed tumor progression at distant sites. Immunofluorescence staining revealed enhanced DC maturation and increased T lymphocyte infiltration within the tumor. CFIN-mediated delivery of NLG919 also inhibited IDO-1, reducing the immunosuppressive TME and improving proinflammatory cytokine expression. While the previous example focused on targeted drug delivery, CFIN effectively harnesses photothermal properties to induce ICD and promote an immune response against cancer cells, exemplifying the versatility of CCM-coated nanoparticles.

In a remarkable advancement in breast cancer treatment, Pan et al*.* [66] combined a CCM coating with a nanoscale metal–organic framework (MOF) core loaded with photosensitizers. This innovative strategy combined photodynamic therapy (PDT) with chemotherapy for enhanced efficacy. The MOF core, containing Fe-tetrakis (4-carboxyphenyl) porphyrin and loaded with tirapazamine (TPZ), exhibited acid-responsive properties. The platform, termed PFTT@CM, featured a hydrodynamic diameter of 201 nm and a zeta potential of − 44.22 mV. The TPZ loading efficiency was determined to be 27.1 ± 7.4%. The CCM coating facilitated immune evasion and tumor retention. Upon reaching cancer cells through endocytosis, the nanoparticles decompose within lysosomes, releasing Fe3 + ions that catalyze the conversion of endogenous hydrogen peroxide (H_2_O_2_) into highly reactive hydroxyl radicals (•OH) while depleting glutathione in the TME. This modulation induced ferroptosis, a specific form of cell death, and enhanced PDT by increasing oxygen levels in the TME, leading to cancer cell apoptosis. PFTT@CM exhibited therapeutic effects of approximately 19.7% (ferroptosis), 43.8% (PDT), and 22.9% (TPZ-based chemotherapy) against MDA-MB-231 breast cancer cells at a concentration of 100 μg/mL (Fig. 4B). Hypoxia activation of TPZ within cancer cells was confirmed. The combined therapeutic approach demonstrated superior anticancer efficacy compared to individual modalities. In vivo experiments in tumor-bearing nude mice showed that PFTT@CM preferentially accumulated at the tumor site, with significant fluorescence intensity observed even 96 h after injection. This synergistic treatment led to complete tumor suppression for 18 days following a single administration.

**Fig. 5:**
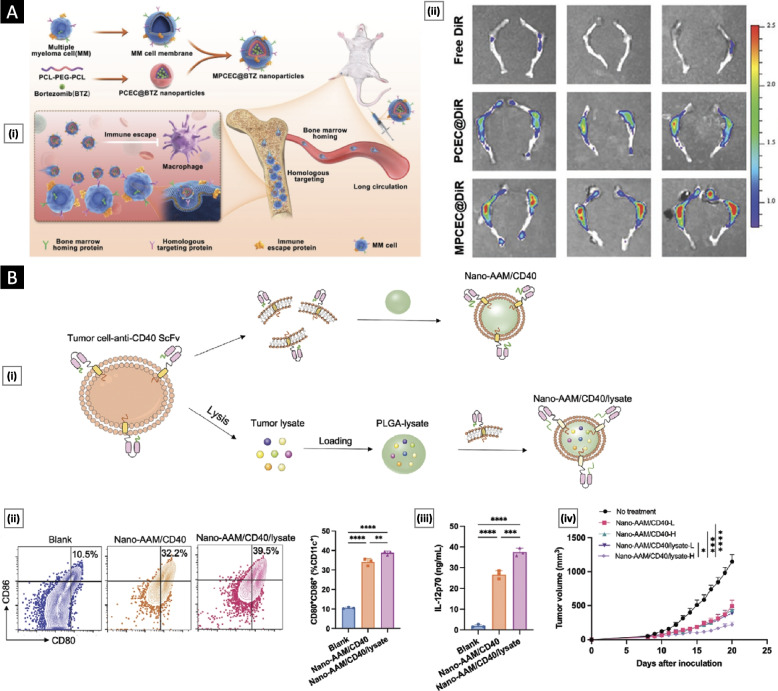
**A** Targeted therapy of multiple myeloma based on bone marrow homing. Subfigure (i) shows a schematic of cell membrane-coated polymeric nanoparticles for the treatment of multiple myeloma. Subfigure (ii) shows the ex vivo fluorescence imaging of the femur tissues at 48 h postinjection of DiR-loaded MPCEC nanoparticles in a 5 T multiple myeloma murine model. Adapted with permission from [68], (Copyright Wiley–VCH, 2022). **B** Genetically engineered antibody-anchored tumor cell membrane as nanovaccines. Subfigure (i) shows a schematic illustration of the design of the nano-AAM/CD40 and nano-AAM/CD40/lysate. Subfigure (ii) shows the flow cytometry analysis of in vitro DC maturation following different treatments. Subfigure (iii) represents ELISA analysis of IL-12p70 production by DCs without and with treatments. Figure (iv) represents the mean tumor growth curve of MC38 tumors in mice after different treatments. Here, *p* < 0.05, **p < 0.01, ***p < 0.001, ****p < 0.0001. Adapted with permission from [69], (Copyright Wiley–VCH, 2023)

To tackle the formidable challenge posed by multiple myeloma (MM), a hematological malignancy known for its aggressive nature and impact on patients' lives, Qu et al*.* [68] developed a novel approach by utilizing the phenomenon of "bone marrow homing," wherein MM cells migrate and return to the bone marrow for survival and proliferation. They fabricated bortezomib (BTZ)-loaded polymeric nanoparticles and further coated them with the MM cell membrane through physical extrusion, forming MPCEC@BTZ nanoparticles. BTZ is a proteasome inhibitor and an established first-line treatment for MM. This platform exhibited remarkable antitumor efficacy in a systemic orthotopic transplantation MM model. The success of this approach can be attributed to the bone marrow localization facilitated by the presence of bone marrow-specific protein markers, including CD44, CD147, CXCR4, and CD138, on the MM cell membrane. The MPCEC@BTZ nanoparticles effectively delayed tumor progression within the BM, improved overall survival rates, and reduced systemic side effects without causing histological toxicity in major organs (Fig. 5A). This innovative platform holds great promise for enhancing the treatment outcomes of MM and improving patient well-being.

In an interesting study, Li et al*.* [69] engineered an antibody-anchored membrane (AAM) nanovaccine by incorporating anti-CD40 single-chain variable fragments (scFv) into tumor cell membranes. This process involved recombinant gene expression, leading to the overexpression of anti-CD40 scFv on the tumor cell surface. The resulting membrane, enriched with anti-CD40 scFv, was then coated onto a polymeric core using water-bath sonication and serial extrusion, yielding nano-AAM/CD40 particles with a mean size of 130.3 nm. CD40, a critical tumor necrosis factor receptor superfamily member, is known for its elevated expression on antigen-presenting cells (APCs) such as DCs. The presence of anti-CD40 scFv on nano-AAM/CD40 enabled specific binding to CD40 receptors on APCs, facilitating efficient delivery of the nanovaccine to lymph nodes. This platform resulted in the maturation of DCs, characterized by the upregulation of costimulatory molecules, effective cross-presentation of antigens, and cytokine production. These responses culminated in the activation and differentiation of T cells. In a prophylactic in vivo study, CD40-humanized transgenic mice vaccinated with nano-AAM/CD40 demonstrated complete prevention of tumor growth when challenged with MC38 tumor cells in 50% of the mice for up to 80 days. To broaden the spectrum of tumor antigens targeted, the researchers loaded the polymeric core with tumor lysate, resulting in nano-AAM/CD40/lysate. This modification significantly enhanced the expression of costimulatory molecules and the production of IL-12 while increasing the density of CD8 + tumor-infiltrating T cells. Treatment with a low nano-AAM/CD40/lysate dose extended median survival compared to low-dose nano-AAM/CD40 alone (Fig. 5B). This innovative nanovaccine platform demonstrates versatility in combination therapy for cancer treatment and is characterized by its precise targeting and immunostimulatory properties.

**Fig. 6:**
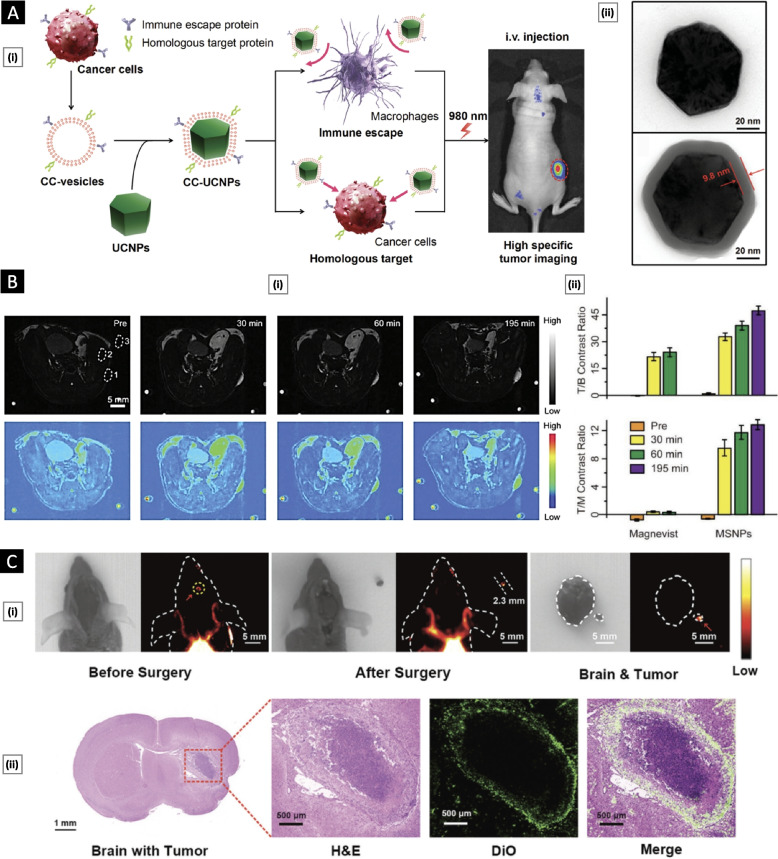
**A** CC-UCNPs for highly specific tumor imaging. Subfigure (i) shows a schematic diagram highlighting the preparation, function, and application of CC-UCNPs. Subfigure (ii) shows TEM analysis of uncoated UCNPs and CC-UCNPs. The cancer membrane was negatively stained with uranyl acetate. Adapted with permission from [54], (Copyright Wiley–VCH, 2016). **B** In vivo tumor visualization using CCM-coated nanoconjugates as MRI contrast agents. Here, subfigure (i) presents the results of T1-weighted magnetic resonance imaging (MRI) along with corresponding pseudocolor images of tumor-bearing mice. The images were captured at different time points following the intravenous injection of MSNPs equivalent to 2.5 µmol of Gd.3+. Subfigure (ii) demonstrates the tumor-to-background (T/B) and tumor-to-muscle (T/M) contrast ratios. Adapted with permission from [72], (Copyright Wiley–VCH, 2019). **C** Imaging and surgical navigation of glioma using CCM-coated lanthanide-doped nanoparticles. Subfigure (i) shows brightfield and near-infrared II (NIR-II) fluorescence images of mice with glioma before and after surgery. Additionally, subfigure (ii) displays an H&E-stained image of the whole brain containing the tumor, alongside a corresponding fluorescence microscope image of the tumor using DiO-labeled CC-LnNPs in green. Adapted with permission from [73], (Copyright Wiley–VCH, 2022)

In the realm of anticancer therapy, the utilization of CCM nanoparticles extends beyond their therapeutic potential. It offers opportunities for early cancer detection and investigation of cancer metastasis through the development of highly sensitive and specific tumor imaging platforms [70, 71]. Rao et al*.* [54] reported the development of CC-UCNPs, a platform that combines CCM with upconversion nanoparticles (UCNPs). UCNPs stand out among fluorescence agents due to their unique optical and chemical properties, including exceptional light penetration depth, narrow emission peaks, high photostability, large Stokes shifts, low toxicity, and minimal background fluorescence. Nevertheless, UCNPs have limitations, such as the lack of targeting ability and susceptibility to the systemic immune system. These challenges were addressed through the application of CCM coating. CCM, obtained via hypotonic lysis, was efficiently coated onto UCNPs using extrusion, resulting in a core–shell-like structure with a hydrodynamic diameter of approximately 100 nm, including a cancer membrane layer of approximately 10 nm (Fig. 6A). The antiphagocytosis properties of CC-UCNPs were assessed in vitro by incubating them with RAW 264.7 cells. Uptake was quantified by measuring the yttrium ion content over various time intervals. CC-UCNPs exhibited minimal uptake compared to their uncoated counterparts. Furthermore, when subjected to a 980 nm NIR laser, these particles displayed exceptional luminescence. In vivo assessments involved using MDA-MB-435 human breast cancer cells, DU145 human prostate cancer cells, CAL27 human squamous cancer cells, and HCT116 human colorectal cancer cells to prepare CC-UCNPs. Using a murine tumor model, the study demonstrated highly efficient targeting when the CCM source cell matched the host tumor. Comprehensive biosafety evaluations through blood biochemistry, hematology tests, and histological analyzes revealed no significant alterations or organ damage, confirming the platform's excellent biocompatibility.

Yi et al*.* [72] introduced MSNPs, a biocompatible nanostructure platform for high signal-to-noise ratio MRI imaging. These MSNPs consist of self-assembled NaGdF4 and CaCO3 nanoconjugates encased within a HeLa cell membrane. In MRI, T1 and T2 relaxation times are crucial for image quality. T1 agents enhance signal intensity in T1-weighted images, while T2 agents do so in T2-weighted images. MSNPs are unique because their T1 source, Gd^3+^ ions, is spatially confined, initially resulting in an "OFF" MRI signal. However, when exposed to a slightly acidic TME, embedded CaCO_3_ nanoparticles generate CO_2_ bubbles, creating an "ON" MRI signal. In vivo experiments on mice with tumors compared the performance of MSNPs with that of the commercial MRI contrast agent Magnevist® (gadopentetic acid). Before MSNP injection, the tumor site appeared dark, but 30 min after injection, it began to illuminate. Approximately 195 min postinjection, the tumor site exhibited significant contrast enhancement, achieving a tumor-to-background ratio of approximately 48, effectively illuminating the entire tumor. Notably, MSNPs outperformed Magnevist®, primarily due to their pronounced tendency to accumulate within tumors. Detailed analysis of specific regions of interest revealed that the tumor-to-muscle signal ratios were approximately 61.6 times higher for MSNPs than for Magnevist® (Fig. 6B).

Liu et al*.* [74] developed a CCM-coated nanosystem called ZGM to enhance metabolic glycan labeling for tumor diagnostic imaging. By incorporating a MOF-azidosugar complex (ZIF-8-Ac4GalNAz), this platform selectively targets homotypic cancer cells through receptors for cell-specific glycan labeling. ZGM cellular internalization occurs through cholesterol-dependent endocytosis, with efficient release from lysosomes due to the "proton-sponge" effect. Notably, ZGM achieved significant metabolic glycan labeling within 12 h without preincubation. The CCM coating protected against macrophage phagocytosis, extending blood circulation and enhancing labeling. In vivo experiments demonstrated ZGM's capability to visualize multiple tumor cell-selective glycans in homotypic tumors, particularly distinguishing between breast cancer subtypes, including the triple-negative and luminal A subtypes. This selectivity holds clinical promise for precise cancer subtype diagnosis.

In a recent study, Wang et al*.* [73] leveraged membrane fragments derived from brain tumors to improve brain tumor resection accuracy using lanthanide-doped nanoparticles (LnNPs). These coated nanoparticles, termed CC-LnNPs, exhibited fluorescence in the near-infrared-IIb window (NIR-IIb, 1500–1700 nm). This study aimed to address the challenges associated with low spatial resolution and limited permeability of the blood‒brain barrier (BBB). Homotypic interaction between the coated nanoparticles and brain tumor cells assisted in crossing the BBB. CC-LnNPs exhibited several advantages, including higher temporal and spatial resolution, improved stability, and lower background signals than the clinically approved imaging agent indocyanine green. Consequently, the boundaries of brain tumors could be visualized more clearly. By leveraging NIR-IIb fluorescence as a guide, the researchers successfully visualized and precisely resected glioma tissue located approximately 2.3 mm within the brain (Fig. 6C).

## Extracellular vesicles

### Understanding CCM

EVs are nanosized vesicles enclosed by a lipid bilayer that are actively released into the extracellular milieu by various eukaryotic cells, including cancer cells and other pathological cell types [75]. They can be detected in various somatic fluids, including blood, urine, bile, saliva, breast milk, and cerebrospinal fluid [76, 77]. When initially discovered in 1967, EVs only function in eliminating cell debris. However, as research has progressed, their importance as imperative bidirectional mediators of cell-to-cell/cell-to-microenvironment signaling in various biological processes has been established [78]. EVs are usually produced in response to intracellular and extracellular stress, such as platelet activation, pH changes, hypoxia, complement protein exposure, irradiation, chemotherapy, and necrosis [79]. The International Society for Extracellular Vesicles emphasizes that EVs should be categorized according to their physical characteristics, surface protein markers, and/or cell source. The most widely applied nomenclature (based on biogenesis and size distribution) classifies EVs into three subtypes: exosomes (30–150 nm), microvesicles (100 nm-1 μm), and apoptotic bodies (1–5 μm) [80]. The following section will exclusively focus on exosomes and microvesicles, as the applicability of apoptotic bodies is limited.

Exosome formation begins when the endosome membrane folds inward, creating a multivesicular endosome. This endosome combines with the cell membrane and releases fully formed exosomes into the extracellular space through exocytosis [81]. In contrast, microvesicles are produced by the outward budding of the plasma membrane. Both vesicles lack functional nuclei, rendering them unable to replicate independently [82]. The ‘endosomal sorting complex required for transport’ protein plays a crucial role in their formation. Based on their biogenesis method, it is anticipated that these EVs contain the same membrane proteins and lipids as the source cell and thus can accurately represent the cell's condition without direct access to it [83]. EVs are enriched with diverse biomolecules, such as nucleic acids, proteins, lipids, and metabolites, that are derived from source cells. To ensure the reliability of using EVs in biomedicine, a validation approach for EVs that involves the verification of specific protein markers is recommended. In the validation process, it is suggested to include at least one membrane protein marker, one cytosolic protein marker, and one non-EV protein marker [84]. To establish the specificity of EV-associated proteins, excluding proteins originating from the nucleus, mitochondria, endoplasmic reticulum, and Golgi complex is essential. These intracellular proteins can serve as negative control markers during the validation process [85]. By adhering to this recommended approach, researchers can ensure the accurate characterization and identification of EV proteins [86]. Most research has identified small noncoding RNAs (specifically microRNAs) as the predominant EV cargo. Isolated EVs may typically comprise hundreds of microRNA species in varying concentrations, all of which play crucial roles in intercellular communication. The standard RNA profiles for EVs collected from different fluids/tissues are available in databases such as ExoRBase [87], exRNA atlas [88], and miRanda [89].

### Role in cancer development

**Fig. 7:**
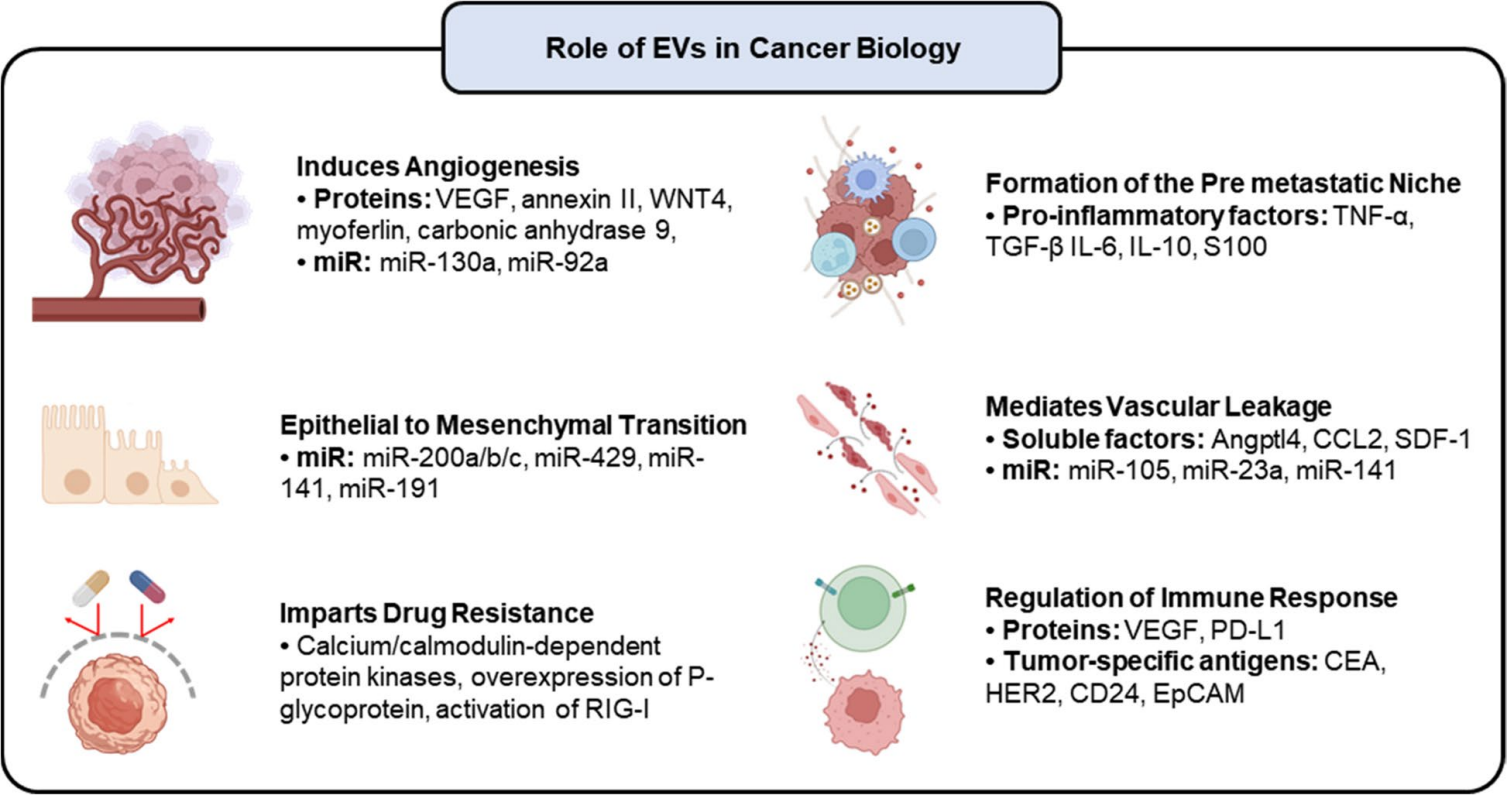
Overview of the role played by tumor-derived EVs (and their cargo) in cancer biology

The intercellular communication facilitated by EVs is paramount for tumorigenesis and metastasis. Multiple studies have demonstrated that the cargo transported by tumor-derived EVs accurately mirrors the dynamic changes occurring within tumor cells throughout various stages, including initiation, progression, invasion, metastasis, and possible relapse [90]. Alteration of tumor neovasculature (enhanced angiogenesis and vascular leakage), formation of premetastatic niches, and modulation of the immune response and drug resistance are some of the critical areas where EVs play a significant role [91]. Figure 7 provides a schematic overview of the role played by EVs (and their cargo) in cancer biology.

Tumor-derived EVs play a crucial role in cancer biology by transferring cargo molecules that contribute to tumor progression and alter the phenotype of recipient cells. One key aspect influenced by EVs is vascular growth, facilitated by activating endothelial cells (ECs). This activation is primarily attributed to vascular endothelial growth factor (VEGF) [92]. Additionally, several EV proteins have been identified to promote angiogenesis, including carbonic anhydrase 9 [93], annexin II [94], myoferlin [95], and wnt4 [96]. In addition, extensive research has been conducted on EV miRNAs, specifically miR-130a and miR-92a, to elucidate their involvement in tumor angiogenesis [97].

The development of premetastatic niches can be facilitated by EVs, which may alter cellular signaling, weaken interendothelial junctions, and increase vascular permeability [98]. Tumor-derived soluble factors such as Angptl4, SDF-1, and CCL2 are carried by EVs and promote vascular leakage, facilitating metastasis. Notably, hypoxic tumor cells release more EVs than their normoxic counterparts, which are regulated by hypoxia-inducible factors (HIFs) [99]. Among various HIF isomers, HIF-1α and HIF-2α are the most well studied [100, 101]. They bind to hypoxia response elements in the promoters of target genes and regulate the expression of genes involved in vesicle biogenesis, trafficking, and release [102]. EVs released under hypoxic conditions contain various constituents, including VEGF [103], the long noncoding RNA CCAT2 [104], and some noncoding RNA miRNAs (miR-25-3p and miR-9) [105], which directly influence the proliferation and migration of ECs. Additionally, miR-23a, miR-92-3p, miR-103, miR-181c, and miR-105 are enriched in these EVs and collectively contribute to the suppression of genes involved in maintaining vascular integrity, ultimately leading to vascular leakage [106]. Furthermore, cancer cells secrete interleukin 3 (IL-3), stimulating ECs to secrete EVs that further promote neovessel formation [107]. The development of premetastatic niches is also aided by tumor-derived EV stimulation of angiogenesis, upregulation of inflammatory chemicals, and suppression of immune responses [108]. These EVs also influence the migration of myeloid cells and contribute to the formation of premetastatic sites by controlling the levels of proinflammatory molecules. Factors and proteins such as TNF-α, transforming growth factor-β (TGF-β), IL-6, IL-10, VEGF, S100, and integrins play essential roles in this complex process [109, 110]. Moreover, EVs exert regulatory effects on DCs, stimulating CD8 + T cells, altering pH levels, and impairing antigen cross-presentation abilities [111]. Remarkably, EVs have the potential to stimulate the immune system and induce an antitumor immune response through the presentation of tumor-associated antigens (EA, HER2, mesothelin, CD24, EpCAM, etc.) to immune cells, particularly cytotoxic T cells [112–114].

miRNAs such as the miR-200 family, miR-23a, and miR-92a-3p are highly concentrated in EVs produced by cancer cells and play a role in triggering epithelial-mesenchymal transition (EMT) in epithelial cells. [115]. EMT is closely connected to several proteins linked to EV-mediated EMT, including HRAS, flTF III, and TGF-β [116–118]. EMT prominently generates CAFs that further secrete factors synergizing with tumor metastasis, such as IL-1β, IL-6, IL-8, and matrix metalloproteinases [119]. In this context, generating CAFs through EMT contributes to establishing a premetastatic niche by cancer cell-derived EVs, as they interact with and prime cells from distant organs. Furthermore, CAFs release proinflammatory cytokines that suppress the normal function of immune cells, maintaining a protumorigenic environment.

Moving forward, the impact of EVs on drug resistance is a critical aspect to consider. Tumor-derived EVs have been identified as key contributors to the development of drug resistance, operating through two distinct pathways: the activation of calcium/calmodulin-dependent protein kinases and the Raf/MEK/ERK kinase cascade [120]. Multiple cancers have been reported to exhibit multidrug resistance due to EV-mediated transfer of P-glycoprotein [121], multidrug resistance-1 [122], and ATP-binding cassette subfamily B member 1. These EVs further contribute to drug resistance by sequestering cytotoxic drugs, effectively reducing their concentration at target sites. Moreover, they serve as decoys by transporting membrane proteins and capturing monoclonal antibodies designed to target cancer cell surface receptors. Finally, it is worth noting that EVs derived from resistant cancer cells can transmit messenger proteins that induce drug resistance in sensitive cells [123, 124]. This interconnected network of EV-mediated processes underscores the multifaceted role of EVs in cancer progression and drug resistance.

### Isolation techniques

Based on the abovementioned explanation that highlights the crosstalk between cancer cell EVs and components of the TME, it is evident that EVs hold tremendous potential for manipulation as tools against cancer itself. Before planning any biomedical applications, an optimal and consistent method to isolate EVs from cancer cells must be employed. Rapid isolation time (preferably under 1 h), high retrieval efficiency with purity (maximum EV yield with minimal protein/free nucleic acid contaminants), and affordability are some of the desirable characteristics of isolation methods [125]. For viable clinical translation, the method should additionally be able to handle high sample volumes and be compatible with automation. An overview of some prominent EV isolation techniques and their respective advantages/disadvantages is discussed in Table 1.

**Table 1:** Techniques for EV isolation

Technique *(sample volume and isolation time)*	Schematic Overview	Advantages	Limitations	Ref
Differential ultracentrifugation *(1.5 mL-25 mL, 3 h-9 h)*		• Minimal reagents, consumables, expertise needed • Suitable for large volumes • Chemical-free, EV-friendly • Reliable reproducibility	• Costly equipment • Low throughput • Elevated protein contamination • Cross-contamination risk • Sterility concerns	[126–128]
Density gradient centrifugation *(1.5 mL-25 mL, 2 h-40 h)*		• High purity (gold standard) • Enables specific EV subpopulation isolation • Affordable reagents	• High equipment cost • Time/labor intensive • Possibility of viral particle contamination (from sucrose) • Significant sample loss	[129–131]
Ultrafiltration *(10 μL-150 mL,* < *2 h)*		• Simple, highly versatile • Rapid, purity-enhancing • Suitable for large samples	• Limited filter lifespan (clogging risk) • Non-EV protein contamination • EV distortion	[132–134]
Size exclusion chromatography *(200 μl-20 ml, 1 min/mL)*		• Rapid isolation • Preserves EV integrity • Prevents aggregation • High purity, reproducible	• Low yield • Low sample volume (not more than 2–5% of the column volume) • Requires EV concentration for downstream applications	[135–137]
Solubility precipitation *(50 μL-10 mL, 30–120 min)*		• Quick process • Kit use reduces labor/equipment needs • Maintains physiological pH	• Costly reagents • Non-EV protein contamination • Poor purity (presence of undifferentiated EV subtypes)	[138–140]
Immunoaffinity-based capture *(10 μL-10 mL,* < *2 h)*		• High purity, reproducibility • High specificity to EV subpopulations • Short processing time	• High reagent costs • Require prior knowledge of exosome tags • Functional loss without antibody detachment	[141–143]
Microfluidic devices *(Variable, 1–15 μL/min)*		• Low sample volume and minimal consumables • Rapid isolation with high purity • Real-time control, automation option	• Requires use-specific design that increases cost • Low throughput	[144–146]

After the successful isolation of EVs, it becomes imperative to comprehensively characterize their clinically relevant attributes. Initial assessments encompass the determination of size and morphology, which can be accomplished through scanning electron microscopy and atomic force microscopy. However, for precise quantification of EV concentrations, specialized methodologies such as scanning ion occlusion sensing or nanoparticle tracking analysis are essential [147, 148]. To gain insight into the composition of EVs, including their associated proteins and nucleic acids, targeted proteomic and transcriptomic analyzes are indispensable [149]. The following subsections will focus on exploiting EVs as tools against cancer.

### Therapeutic applications

Bioengineered EVs are excellent templates for developing advanced cancer nanomedicines. Their intrinsic origin and structural attributes endow them with distinct tumor-targeting characteristics, including augmented systemic circulation and improved tumor penetration, owing to their favorable immunogenic profile and EPR effect, respectively. Furthermore, their capacity for deep tumor penetration arises from their deformability and mechanical flexibility. In addition, they exhibit selective cellular internalization due to the presence of glycans and membrane-soluble ligands that specifically interact with cancer cells [150]. Isolated/purified EVs can be directly used after loading with appropriate exogenous cargo. Small molecule anticancer or drug-loaded nanoparticles can be incorporated into EVs by coincubating them with donor cancer cell lines. While this active loading method is simple, the final loading efficiency is heavily influenced by the physiochemical properties of the drug/drug-nanocarrier system and protocol parameters, which demands extensive optimization [151]. EVs can be passively loaded using saponin-based permeabilization, electroporation (ideal for siRNA or miRNA loading), and mechanical methods, such as freeze‒thawing, sonication, and extrusion [152].

Proper membrane modification strategies can exponentially increase the therapeutic potential of cancer cell-derived EVs. Smyth et al*.* [153] reported the first successful conjugation of ligands to the surface of exosomes (derived from mouse 4T1 cells) using copper-catalyzed azide-alkyne cycloaddition (click chemistry). This study laid the foundation for exploring click chemistry in EV research due to its rapid reaction times, high specificity, and compatibility in aqueous buffers. Subsequently, Jia et al*.* [154] reported the click chemistry-based conjugation of neuropilin-1-targeted peptide to the membrane of exosomes loaded with superparamagnetic iron oxide nanoparticles and curcumin. With the aid of surface-conjugated peptides, the exosomes smoothly crossed the BBB and provided lucrative results for targeted imaging and therapy of glioma. In a different study, azido-modified exosomes obtained from MDA-MB-231 cells were labeled with azadibenzylcyclooctyne fluorescent dyes and applied to examine the in vivo pharmacokinetics of exosomes in MDA-MB-231 tumor-bearing mice [155].

In addition to click chemistry, various noncovalent membrane modifications have been reported with interesting applications. Tamura et al*.* [156] reported positively charged exosomes with enhanced targeting efficiency prepared by electrostatic interactions between negatively charged exosome membranes and cationized pullulan. Sato et al*.* [157] developed an exosome-liposome hybrid by fusing exosomes with antibody- or peptide-functionalized liposomes via a freeze‒thaw method. The hybrid demonstrated active targeting ability while preserving exosome functionality. Recently, artificial “chimeric” exosomes prepared by combining integrated cell membrane proteins (isolated from cancer cell EVs) with synthetic phospholipid bilayers have been extensively reported as novel platforms with high drug-loading capacity and EV-specific functionality [158]. The following section discusses some recent applications of tumor-derived EVs in cancer therapy.

**Fig. 8:**
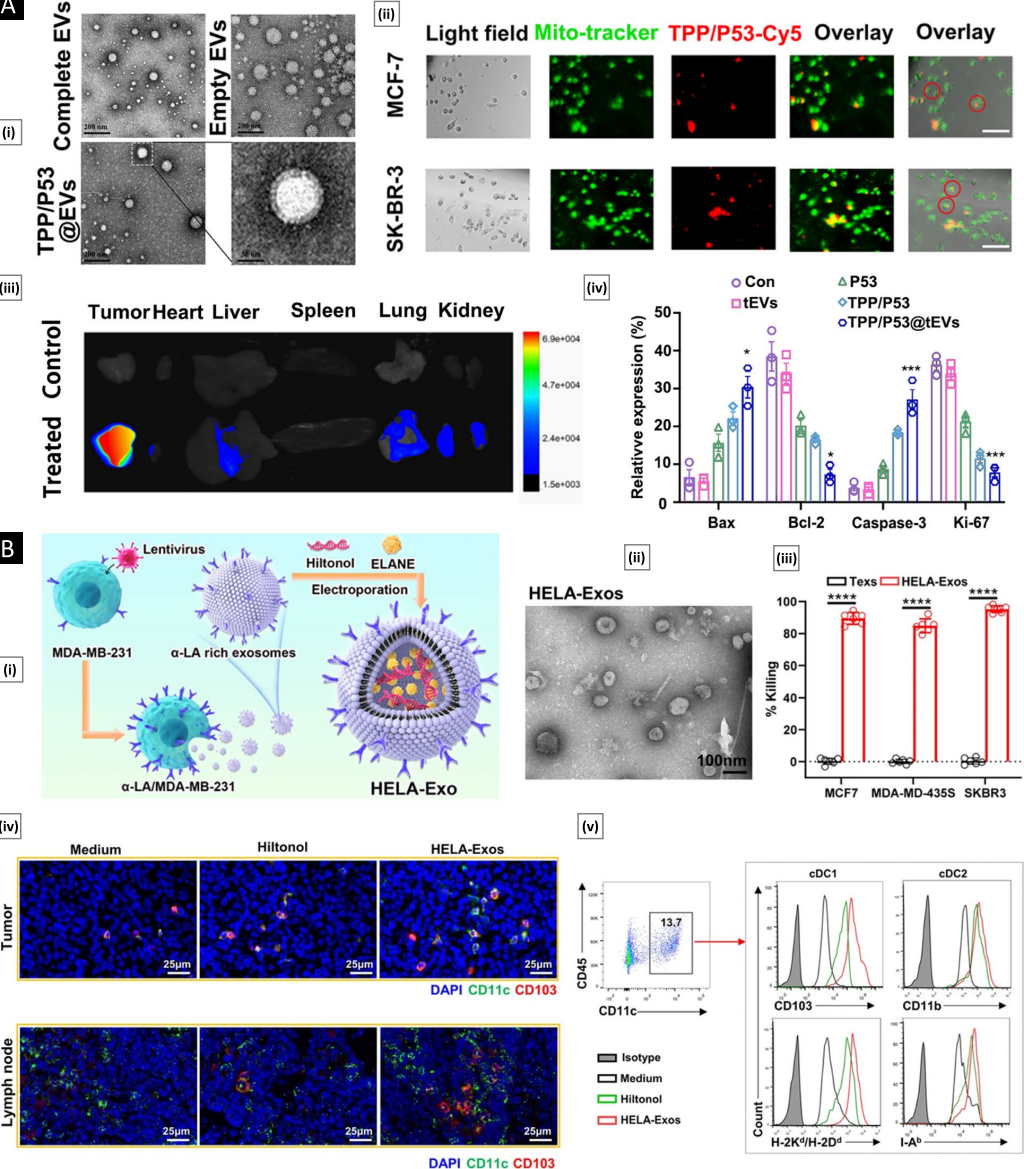
Tumor-derived EVs in cancer therapy. **A** TPP/P53@EVs for treating breast cancer. Subfigure (i) displays the morphological features of the EVs in both unloaded and loaded states. Subfigure (ii) demonstrates the tumor cell targeting ability and mitochondrial localization capacity of TPP/P53@EVs using Cy5 labeling and immunofluorescence imaging. Subfigure (iii) reveals the in vivo biodistribution of TPP/P53@EVs through ex vivo fluorescence imaging. Subfigure (iv) depicts the in vivo toxicity assessment of TPP/P53@EVs through the expression analysis of specific antigens, namely, Bax, Bcl-2, Caspase-3, and Ki-67. Adapted with permission from [159] Copyright Elsevier, 2022). **B** HELA-Exos as an in situ DC-primed vaccine for breast cancer. Subfigure (i) presents a schematic detailing the preparation process. Subfigure (ii) presents TEM images of the HELA-Exos, allowing for observation of their morphological characteristics. Scale bar: 100 nm. Subfigure (iii) displays the results of calcein-AM fluorescence assays used to evaluate the HELA-Exo in vitro killing capabilities against MCF7, MDA-MB-435S, and SKBR3 cells. Figure (iv) illustrates the intratumoral accumulation of cDC1s/CD8 + T cells resulting from HELA-Exo treatment. Subfigure (v) shows the results of a flow cytometry-based analysis of CD11c + DCs. Adapted with permission from [160], (Copyright Springer Nature, 2022)

Jiao et al*.* [159] reported the targeted delivery of exogenous recombinant P53, an apoptosis-inducing protein, using EVs derived from breast cancer cells. This approach involved conjugating P53 proteins with a mitochondria-targeting triphenylphosphonium (TPP) derivative. The TPP/P53 conjugate was loaded into EVs through electroporation, resulting in the creation of TPP/P53@EVs. TEM imaging revealed that unaltered EVs had a homogeneous and bright white interior, indicative of inherent exosomal proteins and RNAs. Electroporation removed the original cargo of EVs and replaced it with TPP/P53 conjugates, resulting in 2–8 TPP/P53 orbicular particles within each EV. Cellular targeting was assessed using MCF-7 and SK-BR-3 cells incubated with Cy5-labeled TPP/P53@EVs, showing TPP/P53 distribution throughout the cells, with notable concentration at the cell center and periphery, indicating mitochondrial targeting. In vivo biodistribution studies in a 4T1 mammary cancer mouse model demonstrated preferential accumulation of TPP/P53@EVs within tumors, with minimal exposure in the liver, lungs, and kidneys. Once fused with the tumor cell membrane, TPP/P53 proteins are released into the cytosol and subsequently transferred to mitochondria. The presence of p53 led to the downregulation of antiapoptotic proteins (Bcl-2), upregulation of proapoptotic proteins (Bax), and increased calcium levels, promoting mitochondrial outer membrane permeabilization and caspase-dependent apoptosis. Notably, no observable toxicity or side effects were detected during the treatment duration in the animal models, underscoring the system's potential safety and efficacy (Fig. 8A).

Huang et al*.* [160] combined Hiltonol (a TLR3 agonist) and the ICD inducer human neutrophil elastase (ELANE) within exosomes derived from MDA-MB-231 breast cancer cells to promote the activation of type one conventional DCs (cDC1s) within the TME. These exosomes were further modified with α-lactalbumin (α-LA), a breast-specific immunodominant protein, to enhance targeting and immunogenicity. TEM analysis revealed that the resulting HELA-Exos had an average diameter of approximately 113 nm. In vitro assessments using the calcein-AM fluorescence test demonstrated that HELA-Exos effectively induced cell death in MCF7, MDA-MB-435S, and SKBR-3 breast cancer cell lines, resulting in a 90% reduction in cancer cell viability compared to a control group treated with tumor-derived exosomes (Texs) alone. In vivo evaluation of HELA-Exos focused on assessing the accumulation of cDC1s and CD8 + T cells in the TME. Immunofluorescence analysis revealed significantly increased infiltration of DCs, identified by markers CD11c + and CD103 + , in both the TME and draining lymph nodes compared to the group treated with Hiltonol alone. Flow cytometry further confirmed the accumulation of the cDC1 subset within the TME, providing strong evidence of the immunogenic activity of HELA-Exos (Fig. 8B).

Extensive global research is being conducted to improve the antigen recognition site and enhance the immunosuppressive TME. However, the goal of achieving these improvements remains elusive. Addressing this challenge, Taghikhani et al*.* [161] reported a study in which they utilized miRNA-modified tumor-derived extracellular vesicles (mt-EVs) to selectively enhance the maturation and antigen presentation capabilities of DCs. Through careful investigation, specific miRNAs (Let-7i, miR-142, and miR-155) that facilitate the desired therapeutic effects of DC maturation and antigen presentation were selectively loaded into mt-EVs. Various combinations and permutations of miRNAs were tested, and it was discovered that miR-142 exhibited the highest expression levels when analyzed using real-time PCR. To assess DC maturation, flow cytometry analysis was performed. The expression levels of surface molecules such as CD11c, MHCII, CD86, and CD40 on DCs treated with mt-EVs were significantly higher than those observed in DCs treated with tumor-derived EVs (t-EVs) alone. Notably, CD11c MHCII expression reached 80% in the mt-EV-treated group and only 65.3% in the t-EV group. The targeted delivery capability of mt-EVs was also evaluated. The expression of miRNAs was solely observed in tumor tissue and nearby lymph nodes, with no detection in other tissues.

Nguyen et al*.* [162] developed a nanosystem using EVs derived from the CT26 murine colorectal cancer (CRC) cell line loaded with doxorubicin (DOX) to create CT26-EV-DOX nanoparticles. TEM images confirmed that the CT26-EV-DOX nanoparticles had a particle size of approximately 217.9 nm. The researchers evaluated the cytotoxicity of these nanoparticles against various types of cancer cells and observed that they exhibited toxicity specifically toward CT26 cells. In vitro experiments were conducted using both 2D and 3D cell culture models to investigate cellular uptake. In the 2D setup, cells were stained with DAPI, while DOX was visualized by its red color. Confocal laser scanning microscopy (CLSM) images confirmed that CT26-EV-DOX nanoparticles were taken up more readily by their parent cells than other cell types. In the 3D tumor setup, CT26 cell spheroids were used, and CT26-EV-DOX nanoparticles demonstrated efficient penetration into the parent cells within the spheroids.

Conventional nanoparticles undergo rapid clearance by macrophages, particularly Kupffer cells in the liver. To address this challenge, Qiu et al*.* [163] developed a novel strategy utilizing exosome-like nanovesicles (ENVs) derived from exosomes of metastatic breast cancer 4T1 cells. ENVs demonstrated the ability to modify the distribution of nucleolin on the cell surface, thereby suppressing phagocytosis by Kupffer cells. Pretreatment with 4T1 ENVs protected therapeutic drugs against elimination by Kupffer cells, thus enhancing the therapeutic efficacy and minimizing potential adverse effects associated with chemotherapeutic drugs. The underlying mechanism behind this phenomenon was attributed to the translocation of membrane nucleolin from the inner face of the plasma membrane to the cell surface, facilitated by ENVs, along with intercellular calcium fluxes. These events resulted in the modulation of gene expression involved in macrophage phagocytosis. Notably, the researchers observed that mice preadministered ENVs exhibited reduced uptake of DOTAP:DOPE liposomes (DDL) in the liver. Consequently, doxorubicin-loaded DDL was redirected to the lungs instead of the liver, effectively impeding breast cancer lung metastasis.

### Diagnostic applications

In addition to anticancer therapy, the major application of EVs is as biomarkers for cancer diagnosis due to the stability and diversity of their biomolecular cargo (such as proteins, lipids, and nucleic acids). These molecules can reflect the presence and staging of tumors, making EVs valuable diagnostic tools. All cell types in the body shed EVs, and their molecular contents are dependent on the cells or tissues of origin. An ideal diagnostic approach should preferentially identify tumor-specific biomarkers at premetastatic phases via a noninvasive method. In this context, body fluid samples that include circulating exosomes loaded with preserved tumor-associated miRNAs can be a novel biomarker source [164]. However, the specific cargo of EVs is not always correlated with the overexpression of molecules in the cells of origin. It can be affected by microenvironmental conditions such as inflammation and metabolic balance [165].

EVs containing biomarkers such as glypican-1 [166], DEL-1 [167], and survivin [168] help distinguish between benign and malignant diseases in breast cancer. EV-CD47 has also been proposed as a possible biomarker for breast cancer [169]. Alterations in the levels of exosomal protein markers (compared to healthy controls), such as gamma-glutamyltransferase in prostate cancer [93], carcinoembryonic antigen in CRC [170], and NY-ESO-1 in non-small cell lung cancer [171], are some prominent examples for cancer diagnosis. Table 2 discusses several cancer-specific exosomal markers and their application in cancer diagnosis. These exosomes can aid in medical decision-making by providing contextual information during treatment. Overall, EVs' stable and diverse cargo makes them a promising avenue for cancer diagnosis and monitoring.

**Table 2:** Reported exosome protein biomarkers for cancer diagnostic applications

Cancer type	Exosome Protein Markers	Function	Application				Ref
			**Prognosis**	**Diagnosis**	**Therapeutic target**	**Disease monitoring**	
Breast cancer	EpCAM	Involved in tumor progression and metastasis	✓	✓	-	✓	[172]
	HER2	Overexpression leads to more aggressive tumor growth and a poorer prognosis	-	✓	-	✓	[173]
	CA 15–3	Involved in tumor growth and metastasis				✓	[174]
	PKG1 (Protein Kinase G1)	Plays a role in cell proliferation and migration. It also regulates estrogen receptor signaling	-	✓	-	-	[175]
Colorectal cancer	CD 147 (Basigin)	It activates PI3K/AKT, MAPK/ERK, and JAK/STAT signaling pathways to promote tumor development, invasion, and metastasis	-	✓	-	-	[176]
	CEA (Carcinoembryonic Antigen)	Activate recipient cell signaling pathways, especially the EGFR pathway, to promote tumor growth, invasion, and angiogenesis	✓	✓	-	-	[177]
	CD 166/ALCAM (Activated Leukocyte Cell Adhesion Molecule)	Promote tumor growth and metastasis	✓	✓	-	-	[178]
	CD 9	CD9-positive exosomes play a role in the dissemination of CRC	✓	-	✓	✓	[179]
Ovarian cancer	HE4 (Human Epididymis Protein 4)	Modulates EGFR-MAPK signaling pathway to influence cancer cell adhesion, migration, and the growth of tumors	-	✓	-	-	[180]
	Mesothelin	Promote tumor development and metastasis	✓	✓	✓	-	[181]
Lung cancer	EGFR (Epidermal Growth Factor Receptor)	Exosomal EGFR activates downstream signaling pathways to promote tumor growth and metastasis	-	✓	-	✓	[182]
	KRAS	Lung cancer often has KRAS protein mutations, which are implicated in numerous cellular signaling pathways	✓	✓	-	-	[183]
	Rab3D	It activates AKT/GSK3β and induces cancer cell EMT, promoting invasion	-	-	✓	-	[184]
	PSMA (Prostate-Specific Membrane Antigen)	It plays a role in the degradation of folate and is abundantly expressed during various phases of prostate cancer, particularly following a relapse in therapy	-	✓	-	✓	[185]
Prostate cancer	PCA 3(Prostate Cancer Antigen 3)	Regulates cancer cell proliferation, invasion, and metastasis	-	✓	-	-	[186]
	CA 19–9	It is produced due to abnormal glycosylation, a process commonly seen in cancer progression, resulting in the formation of various glycosylated residues	-	-	-	✓	[187]
Pancreatic cancer	MUC1	It suppresses the immune response and promotes tumor growth. MUC1-expressing exosomes increase cancer cell migration, invasion, and angiogenesis	-	✓	-	✓	[188]
	AFP (alpha-fetoprotein)	AFP exosomes transfer oncogenic chemicals to promote tumor growth and invasion and suppress T cells and natural killer cells, which kill cancer cells	-	✓	-	✓	[188]
Liver cancer	ANGPT2 (Angiopoietin 2)	It promotes tumor growth and angiogenesis and is associated with increased aggressiveness, invasion, and tumor metastasis	✓	-	-	✓	[189]

Liquid biopsy is a minimally invasive process that uses biological fluids such as blood, urine, or saliva and provides real-time information and simple sample storage [190]. It examines several elements, including EVs, cells, and circulating tumor DNA [191]. EV liquid biopsy, in particular, has become a potential technique for cancer diagnosis, prognosis, and treatment monitoring because of its stability in circulation. The process includes isolation, purification, and detection of EVs from the liquid biopsy sample. Numerous methodologies are available for comprehensively examining biomolecular constituents within cancer cells. These methods encompass DNA sequencing, which furnishes insights into the genetic attributes of cancer; RNA sequencing, which elucidates the gene expression profiles of cancer cells; and proteomics analysis, which offers a glimpse into the protein expression profiles associated with cancer [192]. Liquid biopsy of EVs offers several advantages over traditional tissue biopsy, including its noninvasiveness, the ability to monitor cancer over time, and the potential for early detection. However, the technique is still in the early stages of development, and further research is needed to validate its clinical utility [193]. The following section highlights some interesting applications of tumor-derived EVs in cancer diagnosis.

**Fig. 9:**
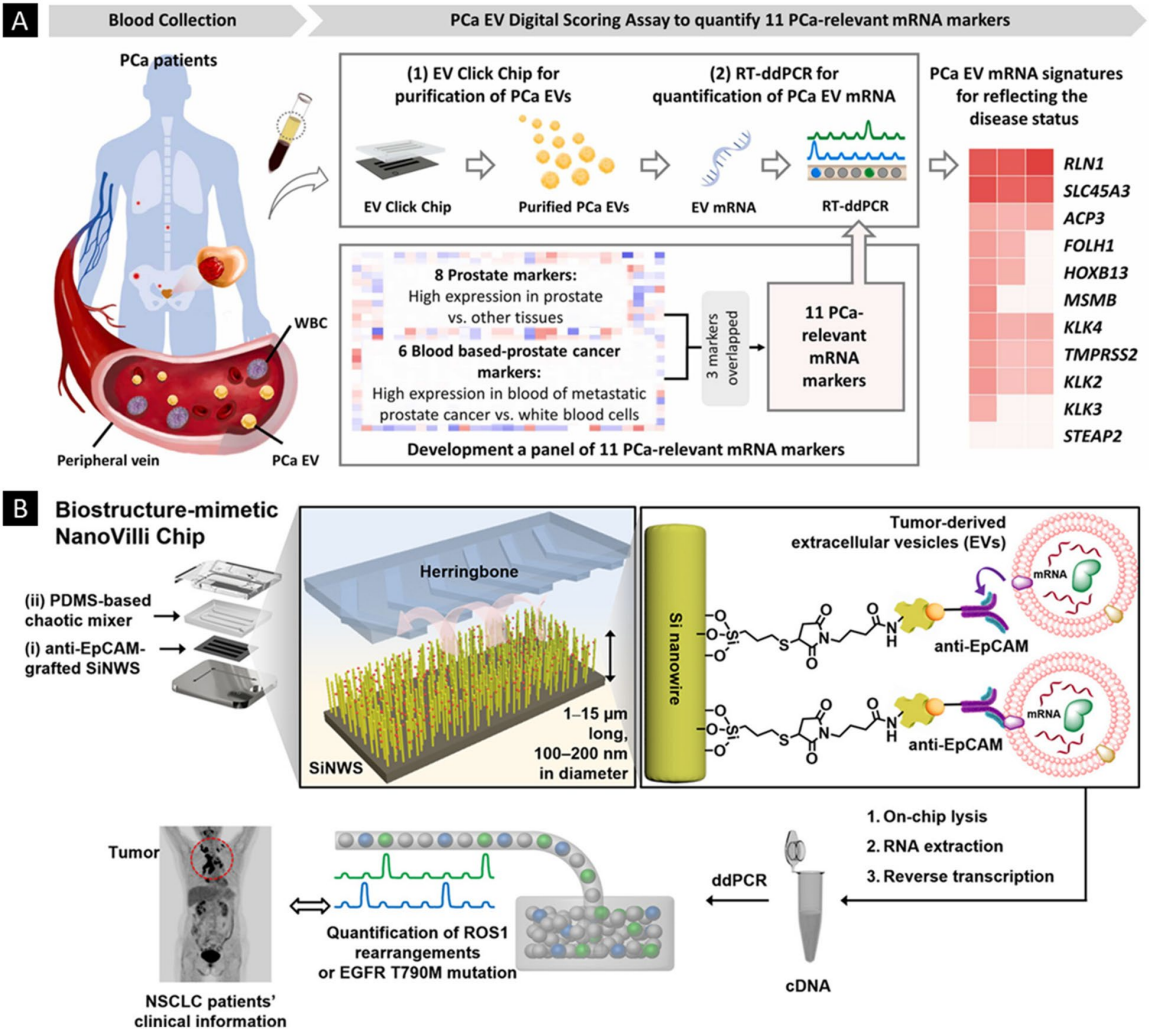
Tumor-derived EVs as a diagnostic agent. **A** Metastasis detection and monitoring of prostate cancer progression using the PCa EV Digital Scoring Assay. Adapted with permission from [194], (Copyright Elsevier, 2023). **B** Schematic illustration depicting the design of the NanoVilli Chip, which takes inspiration from natural biological structures. The chip incorporates densely packed arrays of silicon nanowires that have been grafted with anti-EpCAM antibodies. This design enables the chip to achieve remarkably efficient and reproducible immunoaffinity capture of tumor-derived EVs. Adapted with permission from [195], (Copyright American Chemical Society, 2019)

Wang et al*.* [194] introduced an innovative microfluidics-based device, the EV Click Chip, coupled with a specialized nanosurface, designed to efficiently isolate tumor-derived EVs. These vesicles were explicitly targeted to identify disease-relevant mRNAs, primarily in the context of prostate cancer (PCa). The EV Click Chip facilitated the isolation of pure populations of tumor-derived EVs, overcoming contamination issues by nontumor-derived EVs. Using reverse transcriptase-droplet digital polymerase chain reaction (RT-ddPCR), the researchers developed the EV Digital Scoring Assay (DSA), tailored for rapidly detecting mRNA contents originating from PCa-derived EVs. The assay employed a panel of 11 carefully selected mRNA markers derived from PCa tissue and blood samples, as illustrated in Fig. 9A. The assay's outcome, the Met score, effectively classified PCa in patients as either metastatic or localized. Receiver operating characteristic (ROC) analysis validated the Met score, demonstrating a higher sensitivity (approximately 85%) compared to the commonly used prostate-specific antigen (PSA), with a sensitivity of only approximately 65%. Compared to conventional methods such as ultracentrifugation and the ExoQuick Assay, the EV Click Chip showed superior purification capabilities, even with smaller plasma samples. However, the study acknowledged limitations, such as the need for more appropriate gene candidates specific to EV-based studies. As research into EV cargo continues, the assay holds potential for further optimization by targeting new or evolving disease biology.

Dong et al*.* [195] introduced the nano-Villi chip, inspired by the efficient surface area of intestinal villi, as an innovative method for capturing tumor-derived EVs from limited blood plasma samples. This chip comprises two key components: a silicon nanowire with anti-epithelial cell adhesion molecule (anti-EPCAM) grafting and a polydimethylsiloxane-based chaotic mixer equipped with a microchannel. The herringbone arrangement within the mixer facilitates contact between the anti-EPCAM-grafted nanowire and tumor-derived EVs, enhancing capture efficiency. RNA content from captured EVs was assessed using a Qubit 3.0 Fluorometer and Qubit RNA HS assay, followed by reverse transcription droplet digital PCR. Longer silicon wires significantly improved RNA recovery rates (82 ± 8%) compared to shorter silicon wires (60 ± 6%) and flat silicon substrates (31 ± 1%). The presence of anti-EPCAM was critical for efficient EV capture, primarily targeting EVs with diameters between 30 and 300 nm. The clinically applicable nano-Villi chip demonstrated its utility for quantitatively detecting genetic alterations in non-small cell lung cancer (NSCLC), highlighting its potential in clinical settings (Fig. 9B).

Kang et al*.* [196] introduced an innovative approach termed the extracellular vesicle on demand (EVOD) chip, designed to detect and diagnose lung cancer by isolating cancer-associated exosomes derived from lung cancer cells. This method relies on inverse electron-demand Diels–Alder click chemistry and involves several technical steps. First, a cross-linking agent is employed to immobilize trans-cyclooctene prelinked with primary amines onto a capture surface, creating a three-dimensional isolation structure. Subsequently, tetrazine molecules are attached to the surfaces of the exosomes, either through direct bonding with N-hydroxysuccinimide ester or by conjugation with specific antibodies. The EVOD chip capitalizes on the rapid membrane-bound reaction between trans-cyclooctene and tetrazine within the device, ensuring instantaneous and efficient isolation of exosomes. Following isolation, the exosomes are liberated from the chip by cleaving the disulfide bond with dithiothreitol (DTT). The authors systematically fine-tuned the concentration and flow rate of DTT release to optimize the recovered exosome yield and purity. This precision enhancement in the isolation process holds immense potential for the in-depth study of cancer-associated exosomes. It may usher in new clinical applications in cancer diagnosis and treatment. By offering a rapid and effective EV isolation technique, the EVOD chip opens up new avenues for investigating the role of EVs in cancer and advancing early cancer detection strategies.

In a similar study by Notarangelo et al*.* [197], a novel technique known as nickel-based isolation (NBI) was introduced. NBI utilizes a nickel-functionalized matrix to capture EVs via electrostatic interactions, ensuring EV stability and integrity preservation. Chelating agents release EVs while maintaining their structural integrity for subsequent analysis. NBI demonstrates efficient selective enrichment of heterogeneous EVs within the 50–700 nm size range, providing a time-efficient and cost-effective method. It addresses the common issues of protein contaminants and surface charge fluctuations associated with traditional isolation methods. To overcome challenges related to correlating vesicle size with their cell of origin, the study introduces the concept of EV lineages. These are mixed vesicle populations positive for a cell type-specific marker, indicating a common parental origin. The approach allows for unbiased recovery of different EV lineages, resulting in a homogeneous suspension of polydisperse EV lineages. Additionally, the study presents a new droplet digital polymerase chain reaction assay that eliminates the need for RNA extraction. The platform identified fractions of secreted EVs carrying tumor biomarkers with enhanced sensitivity and accuracy, potentially reevaluating the mutational status in at least 10% of the analyzed patients. This suggests that NBI-ddPCR could be used to infer the presence of specific cell subpopulations that are difficult to detect in tissue biopsies, providing a deeper understanding of tumor heterogeneity and its evolution during therapy.

## Whole cells and tumor lysates

### Understanding cancer vaccines

Vaccines, which prevent disease by training the body’s immune system to rapidly and specifically terminate harmful pathogens, are widely regarded as one of the greatest medical inventions. They have single-handedly contributed to saving countless lives by facilitating the elimination of smallpox and the near eradication of other life-threatening diseases, such as polio and diphtheria [198]. For the past 50 years, the lucrative proposition to develop therapeutic cancer vaccines (TCVs) has been a research hotspot, but most of these efforts have met with unsatisfactory outcomes. While vaccines against human papillomavirus and hepatitis B (cervical and liver cancer, respectively) have received Food and Drug Administration (FDA) approval, the poor clinical translatability of other TCVs can be attributed to the immunosuppressive context of their utilization (suboptimal antigens, lack of immunostimulatory adjuvants, inadequate tumor localization of cytotoxic T-cell lymphocytes) [199].

**Fig. 10:**
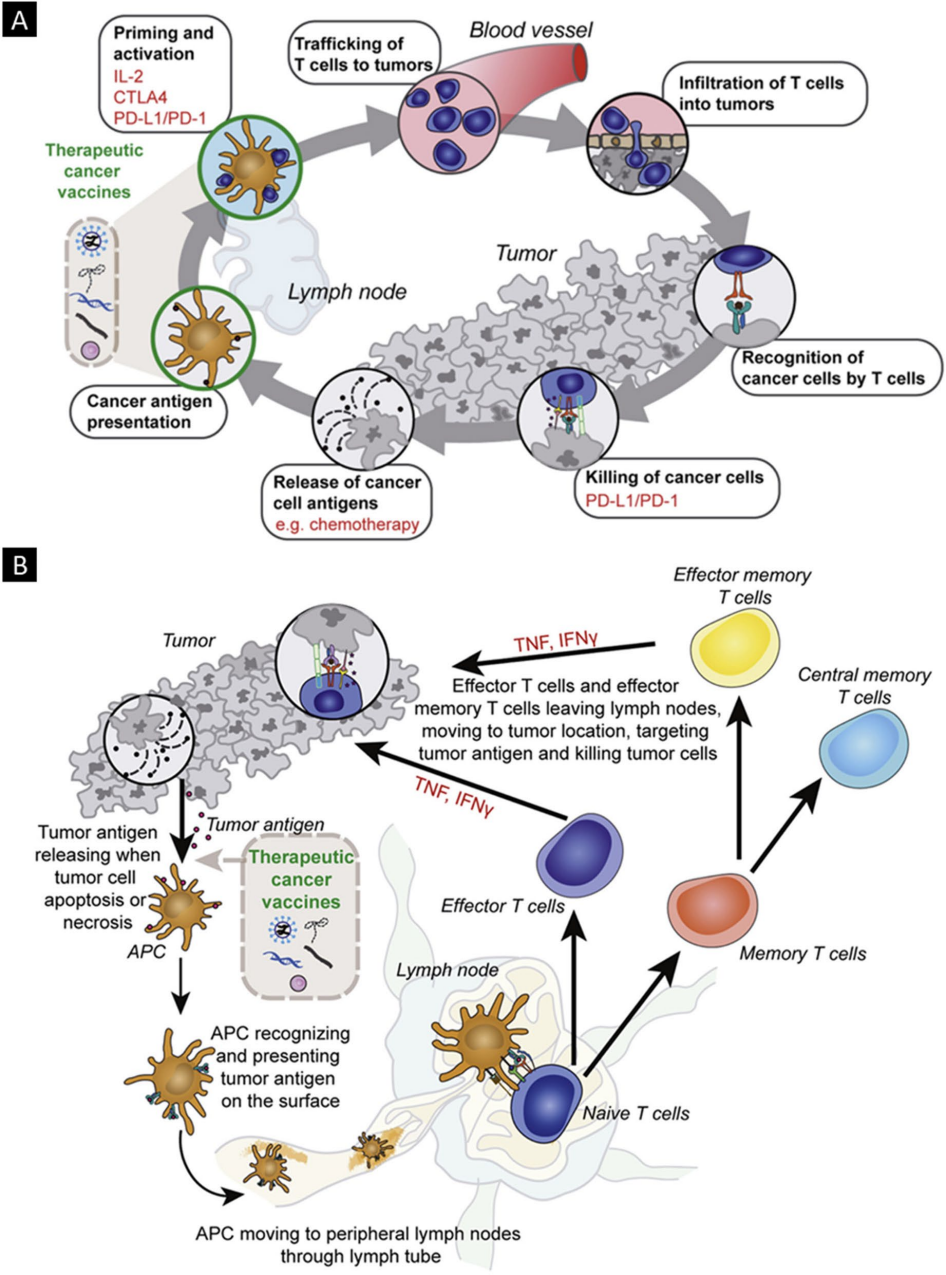
Mechanisms responsible for promoting tumor immunity and generating effective T-cell responses against tumors within the human body. TCVs activate the immune response by presenting cancer antigens to DCs, activating CTLs that provide long-term immunity. **A** Developing immunity against cancer is cyclic and involves both immunostimulatory and inhibitory factors. The cycle consists of seven main steps, starting with the release of antigens from cancer cells and ending with their eradication. TCVs and combination immunotherapy affect particular phases of the cancer immune cycle. In addition, blocking PD-L1 or PD-1 can eliminate the suppression of T-cell-mediated cancer cell death. Vaccines boost the presentation of the cancer antigen. Anti-CTLA4 promotes the priming and activation of antigen-specific T cells. **B** DCs in the TME or peripheral blood interact with tumor antigens, which triggers the immune system to produce antitumor T-cell responses. These antigens are delivered to T cells by activated DCs, which causes effector and memory T cells to be activated and differentiate. Once cancer cells are targeted and destroyed, memory T cells can produce an effective secondary response when subjected to subsequent exposure to the same tumor antigen. Adapted with permission from [199] (Copyright Elsevier, 2021)

The use of personalized antigen sources (especially modified whole cancer cells and TCLs), alone or in combination with appropriate adjuvants, has opened new avenues to develop robust TCVs [200]. Before deep-diving into these novel TCV opportunities, it is vital to comprehend the key elements and immune mechanisms that cause tumor immunity. The main goal of any TCV is to induce a robust antitumor T-cell response. TCVs engage and leverage several facets of cancer immunity, such as the presentation of cancer antigens, priming and activation of T cells, and cancer cell recognition and subsequent elimination [201]. By coadministering adjuvants, both innate and adaptive responses can be triggered simultaneously. Pattern recognition receptors (PRRs), e.g., TLRs, identify and retort pathogen-associated molecular patterns, thereby triggering nonspecific innate immune responses. The transcription factor nuclear factor κB (NF-κB) is then stimulated, leading to enhanced synthesis of cytokines/chemokines and activation of lymphocytes [202]. Finally, TCVs induce an adaptive cytotoxic T-cell lymphocyte-mediated antitumor response by serving as a chemotactic platform to commence immune crosstalk with APCs. TCVs present immunogenic tumor antigens to APCs, leading to recruitment and maturation. These mature APCs, under the influence of key stimulatory signals and cytokines, activate CD8 + T cells. These individual steps generate an enduring immunological memory proficient in constraining tumor growth and preventing relapse/metastasis [203]. A schematic overview of TCV-mediated tumor immunity is depicted in Fig. 10.

### Cell/lysate-derived tumor antigens

**Fig. 11:**
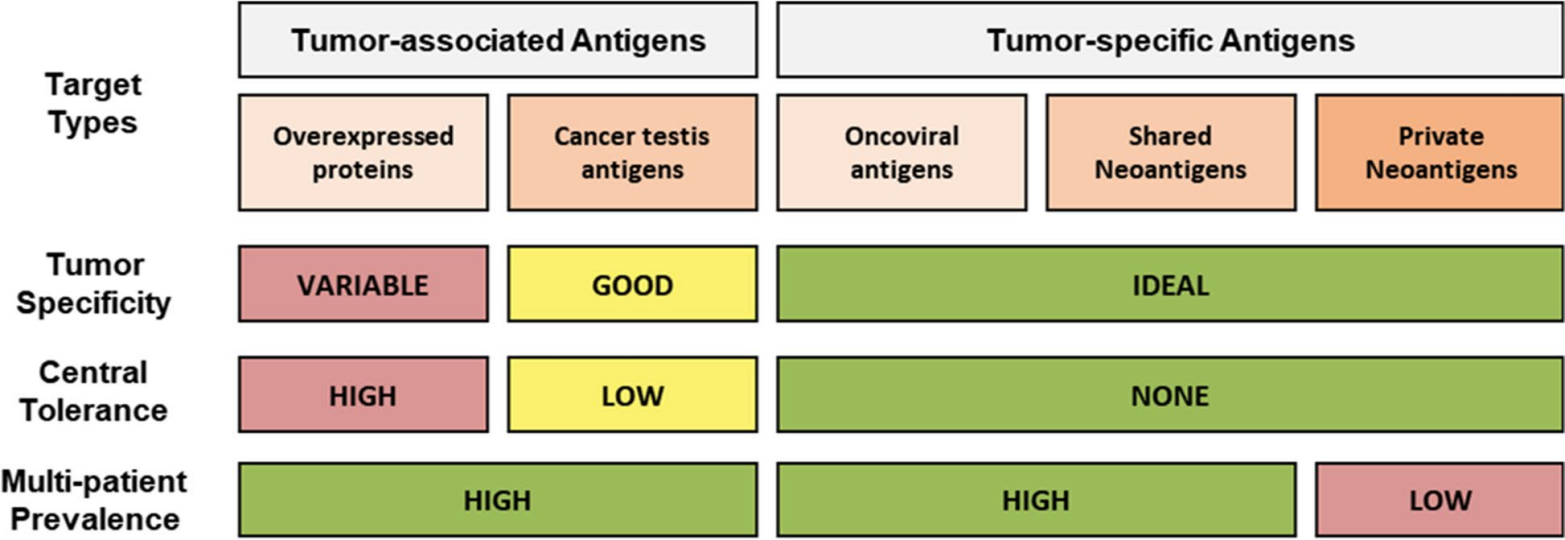
Schematic representation of different tumor antigen sources and their attributes. Recreated with permission from [202], (Copyright Springer Nature, 2019)

Tumor antigen selection is a critical aspect of TCV development. An ideal antigen should be exclusively produced by cancer cells, present on all cancer cells, and be an integral part of cancer cell survival. Utilization of such antigens will decrease the probability of “immune escape” by means of antigen downregulation (thus yielding high immunogenicity) [204, 205]. A single antigen will rarely possess all these attributes. Regardless, tumor antigens are classified into two broad categories: tumor-specific antigens (TSAs) and tumor-associated antigens (TAAs). Different tumor antigen sources and their attributes are highlighted in Fig. 11. TAAs are abnormally expressed “self-proteins” of cancerous cells. Examples of TAAs that have been identified and utilized in TCVs include cancer/germline antigens (e.g., MAGE-A1, MAGE-A3, and NY-ESO-1) [206]; cell lineage differentiation antigens (e.g., tyrosinase, MART-1, prostate-specific antigen) [207]; and overexpressed cancer antigens (e.g., hTERT, HER2, mesothelin, and MUC-1) [208]. While TAAs produce specific cellular and humoral immune responses, their “self-antigen” nature makes them susceptible to central immune tolerance, requiring costimulators or repeated vaccination to amplify the immune response [209]. Since TAAs are also expressed by normal cells, significant efforts are necessary to address any potential risk to normal cells. In this sense, TSAs are superior antigens, as they are absent in normal cells. Oncogenic viral antigens and neoantigens are examples of TSAs. Neoantigens are a subtype of major histocompatibility complex (MHC)-binding peptide originating from cancer-specific mutations [210]. “Shared” neoantigens are common mutations that can be largely identified in a wider range of patients (for particular cancers), making them excellent targets for off-the-shelf TCVs. “Private” neoantigens are rare mutations that are highly patient-specific and appropriate for more personalized vaccine development [211]. It should be noted that neoantigen identification is an expensive and laborious process.

TAAs are self-antigens expressed abnormally in tumor cells, while TSAs are tumor-specific and expressed by oncoviruses or cancer mutations. TSAs are more effective because high-affinity T cells may already be present, while TAAs require a potent vaccine to “break tolerance.” Some TSAs are shared among patients, while others are unique and require personalized therapy. Employing whole cancer cells or TCLs as TCV components is a viable proposition based on the available knowledge. They can supply all prospective antigens, removing the obligation to target the best antigen in a specific tumor. Multiple tumor antigens can be targeted simultaneously, provoking an assorted immune response that avoids antigen loss [212]. “Cellular” vaccines can be prepared using modified whole tumor cells or TCLs generated by irradiation or repetitive freeze‒thawing. Cancer cells can be sourced from the patient (autologous) or an appropriate donor (allogeneic) for use as antigens. Nevertheless, autologous cancer cells impart stronger tumor-specific immunity, as they carry the entire antigenic profile of the patient’s tumor. In theory, tumor cell lines can also be used, but patient-derived primary cells are favored, as their preexposure to the immune system provides additional tumor antigens [204].

### Applications in cancer immunization

Various strategies have been devised to enhance the immunogenicity of these cancer cell-derived components. Using retroviral or adenoviral transduction, cancer cells can be modified to express molecules relevant for immune activation, such as cytokines (especially granulocyte–macrophage colony-stimulating factor), chemokines, or costimulatory molecules that function as adjuvants [213]. By inducing ICD in cancer cells before using them (whole or in lysate form), various damage-associated molecular patterns (DAMPs), such as calreticulin, heat shock proteins (HSP 70/90), pentraxin-3, and uric acid, are released, which synergize with dendritic cell maturation and T-cell responses against the tumor [214]. Theoretically, immunization with antigens derived from cancer cells will undoubtedly expand their therapeutic uses. Nevertheless, their clinical efficacy may be constrained by uncertainty regarding their optimum dose and route of administration [215]. This drawback can be circumvented by using considerately bioengineered delivery platforms, some of which are discussed below.

**Fig. 12:**
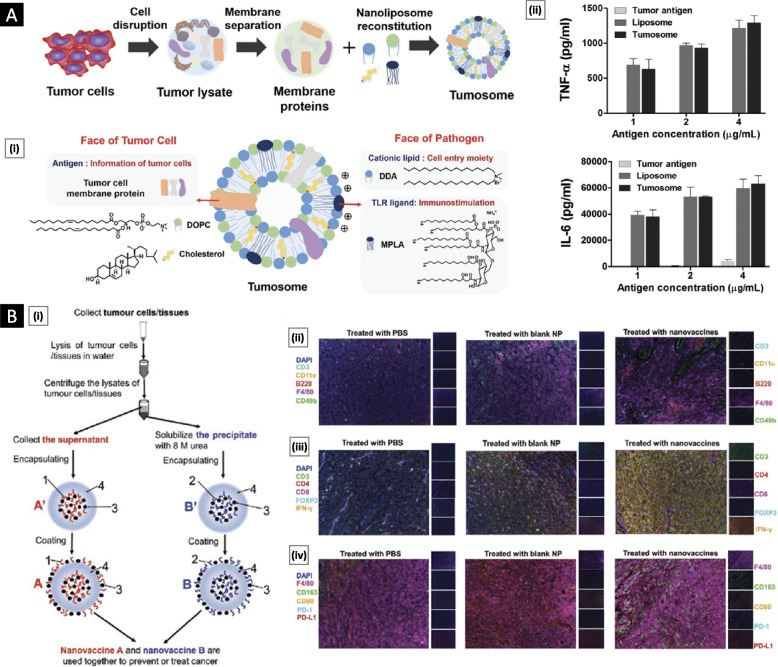
Using cancer cell tumor lysate for immunomodulatory effects. **A** Tumosomes for enhancing antitumor immunity. Here, subfigure (i) illustrates the synthesis of multifaceted Tumosomes with immunostimulant adjuvants and helper lipids. Subfigure (ii) demonstrates immune cell maturation by measuring the levels of proinflammatory cytokines such as TNF-α and IL-6 with ELISA. Adapted with permission from [216], (Copyright Wiley–VCH, 2017). **B** Nanovaccine preparation using whole TCL. Here, subfigure (i) depicts a brief graphic representation of the synthesis of the nanovaccine. Subfigure (ii) shows the activation of adaptive immunity by the activation of DCs, macrophages, B cells, and T cells. Subfigure (iii) shows the activation of innate immunity. Increased levels of CD8 + and CD4 + T cells increase the IFN-γ responsible for tumor death. Figure (iv) shows enhanced macrophage type I expression by investigating F4/80, CD163, CD80, PD-1, and PD-L1 through immunohistochemistry. Adapted with permission from [217], (Copyright Wiley–VCH, 2021)

Noh et al*.* [216] introduced an innovative immunomodulatory nanoliposome system named Tumosome, which combines tumor cell membranes from whole tumor lysates with two lipid-based adjuvants: 3-O-desacyl-4′-monophosphoryl lipid A (MPLA) and dimethyldioctadecylammonium bromide (DDA). MPLA, derived from Salmonella Minnesota, was chosen as a TLR ligand to stimulate key cytokines involved in immune activation, such as IL-6, IL-1β, IL-12, IFN-β, and TNF-α, via the TLR4 receptor. As MPLA and DDA are hydrophobic, they require delivery in the form of an emulsion or liposome. DDA, known for its cationic properties, aids in the uptake of tumor antigens by APCs. Tumosome preparation involves tumor cells obtained from the patient or an allogenic tumor cancer cell line. The study utilized fluorescence techniques to demonstrate enhanced cellular uptake of Tumosomes. Furthermore, the secretion of the proinflammatory cytokines IL-6 and TNF-α was measured via ELISA to assess the potential of Tumosomes to activate and mature immune cells, specifically bone marrow-derived dendritic cells (BMDCs) and bone marrow-derived macrophages. The secretion of cytokines was concentration-dependent (Fig. 12A). Overall, this study underscores Tumosomes' capacity for simultaneous tumor antigen delivery and immune cell activation, suggesting their potential in cancer immunotherapy.

Lin Ma et al*.* [217] reported the development of a cancer vaccine by solubilizing the entire TCL fraction using 8 M urea. This solubilized lysate was then incorporated into a vaccine formulation comprising PLGA, creating a versatile vaccine capable of carrying various tumor antigens. The size of the resulting nanovaccine was meticulously controlled at 300 nm to facilitate efficient delivery into APCs. The tumor was initially fragmented into microsized pieces, followed by centrifugation. The water-soluble component in the supernatant was collected, while the insoluble component in the precipitate was solubilized using 8 M urea. These water-soluble and insoluble components were then loaded into PLGA nanoparticles, leading to the development of nanovaccines A and B, respectively. Using the double-emulsion method and mixing/coating techniques, all essential antigens were loaded inside and on the surface of the PLGA nanoparticles. This nanovaccine exhibited the ability to activate both adaptive and innate immune responses. The study revealed a significant increase in the abundance of various immune cells at the tumor site in the nanovaccine-treated group. Notably, the levels of DCs, macrophages, B cells, and T cells were notably elevated, reflecting the activation of adaptive immunity against cancer. B cells and tertiary lymphoid structures were also observed, suggesting their role in enhancing immunotherapy. Furthermore, nanovaccine treatment resulted in increased levels of natural killer cells and enhanced CD8 + and CD4 + T-cell activity, characterized by elevated cytotoxic interferon-gamma (IFN-γ) secretion. Most macrophages at the tumor site following nanovaccine treatment exhibited the type 1 macrophage (M1) phenotype, known for its effectiveness in eliminating cancer cells. Flow cytometry studies further supported these findings, showing an increase in cytotoxic T cells after nanovaccine treatment (Fig. 12B).

Wang et al*.* [218] employed polydopamine nanoparticles loaded with tumor lysate (TCL@PDA NPs) for cancer immunotherapy. The TCL@PDA NPs demonstrated excellent storage stability and minimal cytotoxicity. Upon uptake by BMDCs, TCL@PDA NPs promoted antigen assimilation and BMDC maturation, leading to enhanced surface molecule expression and the secretion of Th1-related cytokines. In vivo administration of TCL@PDA NPs to mice resulted in significant tumor growth suppression in treatment and prevention models. This was accompanied by increased subpopulations of CD4 + and CD8 + T cells in the spleen and lymph nodes and an increase in the production of memory T cells, providing long-term protection against malignancies. The increased number of CTLs and M1-type TAMs and decreased subpopulation of immunosuppressive cells in the tumor tissues demonstrated the antitumor activity of TCL@PDA NPs. Furthermore, empty PDA NPs demonstrated the ability to modulate DC maturation by enhancing their capacity to express MHC II and secrete Th1-related cytokines, inhibiting tumor development by promoting the production of activated T cells and reducing the subpopulation of MDSCs in tumors.

In a similar study, Wang et al*.* [219] developed a tumor microparticle vaccine. It involved coating live tumor cells with a specialized layer containing epigallocatechin-3-gallate (EGCG) and aluminum (III) (Al(III)). EGCG, a well-known polyphenol with anticancer properties, stimulated macrophage activation by enhancing Th1 cytokines such as TNF-α and INF-γ while reducing immunotolerance-related cytokines such as IL-10. Al(III) is an effective aluminum adjuvant known for eliciting a robust humoral immune response. EGCG and Al(III) were coordinated to form an EGCG/Al(III) complex, which was then coated with inactivated B16 tumor cells, resulting in the TCL@EGCG/Al(III) complex. This coating process was successfully applied to four different types of cancer cells, demonstrating compatibility. Cell viability studies indicated that the process had no adverse effects on cell viability. Furthermore, the study investigated antigen uptake by DCs using microparticles as a delivery system. The results showed that DCs efficiently phagocytosed these microparticles, displaying dendritic structures indicative of their maturation. Flow cytometry analysis quantified antigen uptake and revealed significantly increased uptake by DCs when using the microparticle delivery system compared to soluble antigens. After antigen uptake, BMDCs matured and expressed activation markers, enhancing their capacity to present antigens and prime T cells. Notably, the microparticle delivery system upregulated the expression of CD40, CD80, MHC I, and MHC II on the surface of BMDCs, indicating its role in BMDC maturation. Additionally, it induced the production of Th1-related cytokines such as IL-12p70, TNF-α, IL-6, and interferon-γ, which are crucial for promoting the differentiation of T cells into CD8 + cytolytic T lymphocytes, highlighting its significant potential as a personalized cancer immunotherapy adaptable to different cancer types.

Numerous studies have investigated the role of adjuvants in conjunction with TCL and their synergistic effects in targeting and eliminating tumor cells. Ashrafi et al*.* [220] conducted similar research to explore the impact of propranolol, an adjuvant, when combined with a tumor lysate vaccine in a mouse model of breast cancer. Their study focused on evaluating immune responses and tumor growth. The findings revealed that administering propranolol alongside the vaccine containing TCL derived from the 4T1 breast cancer cell line significantly enhanced lymphocyte proliferation and cytokine production within the TME. Notably, cytokines such as IL-2, IL-10, IL-12, IFN-, and IL-17 were notably increased. Moreover, the propranolol/vaccine combination effectively suppressed tumor growth compared to the group immunized solely with tumor lysate. These results underscore the potential of propranolol as an adjuvant in cancer immunotherapy. The study also suggests that the propranolol/vaccine mixture may induce Th1 cytokine responses, which are recognized for their involvement in antitumor immune reactions. Additionally, the combination of propranolol and tumor lysate vaccine holds promise for expanding IL-17-based therapies in breast cancer treatment.

Shi et al*.* [221] synthesized chitosan nanoparticles decorated with mannose to target DCs. These nanoparticles were loaded with TCL derived from B16 melanoma cells (Man-CTS-TCL NPs). When tested in BMDCs, the system demonstrated significantly higher antigen uptake than other groups, as assessed by flow cytometry. Moreover, the Man-CTS-TCL NPs promoted the maturation of DCs, as evidenced by the enhanced expression of surface markers such as CD80, CD86, and CD40. A cytotoxic T lymphocyte assay was performed to assess T-cell efficacy in tumor-mediated specificity. Splenocytes, as effector cells, were cocultured with B16 melanoma cells, and lysis of the target cells was evaluated at different effector:target (E:T) cell ratios. The results showed that effector T cells obtained from mice immunized with Man-CTS-TCL NPs exhibited a higher efficiency in inducing the CTL response against B16 melanoma target cells, with a lysis rate of approximately 35% compared to the control group's rate of approximately 12%. C57BL/6 mice with B16 melanoma vaccinated with Man-CTS-TCL NPs displayed increased CD8 + T cells in the spleen and elevated expression of IFN-γ.

## Engineered cancer cells and tumor organoids

### Understanding engineered cancer cell lines

Cancer cell lines are cells obtained from cancerous tissues and can proliferate continuously in laboratory conditions, offering a continuous supply of cancer cells for scientific investigations. Cancer cell lines are typically generated by immortalizing cancer cells, which involves introducing genetic changes that hinder or bypass the mechanisms that regulate cell growth and division. Common methods of immortalization include inducing mutations in cell cycle control genes such as TP53, RB1, or PTEN, which usually inhibit uncontrolled cell growth and division [222]. Once immortalized, cancer cell lines can be propagated and maintained in culture indefinitely, making them an invaluable resource in cancer research. They are extensively utilized to scrutinize the biology of cancer, test new cancer treatments, evaluate drug toxicity and effectiveness, and pinpoint prospective targets for cancer therapies [223]. Cancer cell lines offer numerous benefits for scientific research. They can be grown in substantial quantities, enabling experiments and testing on a large scale. They can also be cryopreserved and preserved for long periods, allowing researchers to work with the same cell lines for years or even decades. Furthermore, cancer cell lines are often easier to manipulate than whole animals, which enhances experimental control and reproducibility [224].

During the early 1990s, the National Cancer Institute in Bethesda, MD, introduced a new "disease-oriented" approach for evaluating new anticancer drugs. This involved using a panel of 60 human cancer cell lines derived from nine different types of cancer (brain, colon, leukemia, lung, melanoma, ovarian, renal, breast, and prostate) to perform high-throughput screening of potentially marketable drug candidates [225]. The primary focus was to narrow down the most likely candidates subjected to further preclinical assessment in xenograft models. However, this approach had poor robustness in identifying promising candidate drugs, leading to the adoption of the hollow fiber assay (which involves the implantation of hollow fibers, which are small, semipermeable tubes containing cancer cells into a mouse/rat). The fibers are then exposed to various anticancer drugs to assess their cytotoxicity [226]. The NCI uses only 12 human cell lines for regular preliminary screening before moving on to time-consuming and labor-intensive xenograft experiments for the most promising therapeutic candidates.

Despite widespread efforts to reduce and eliminate animal testing for anticancer drug screening, cancer cell line-based in vitro methods have some critical drawbacks that make them unreliable. These drawbacks can be narrowed down to the extensive genetic/epigenetic changes of cells in culture, lack of tumor heterogeneity (as in primary cancer), and total absence of relevant components constituting the complex TME [227, 228]. Several strategies have been employed to mitigate these limitations. One approach involves utilizing primary cancer cells derived directly from tumor biopsies or surgical specimens obtained from cancer patients. These primary cells closely mirror the biological characteristics of the original tumor, offering a high degree of fidelity to the source malignancy. Another strategy involves the use of genetically modified cancer cell lines. These cell lines are engineered to carry specific genetic alterations, such as the overexpression or knockdown of genes implicated in cancer development and progression. Additionally, they can be modified to express specific cytokines and growth factors known to be present in the TME [229]. Primary tumor cells provide a superior representation of the genetic and phenotypic diversity present in clinical tumor samples. However, the degree of heterogeneity is often not comprehensively defined, rendering the interpretation of experimental outcomes challenging. Even well-defined cancer lines can pose a challenge in screening therapies targeted toward specific oncogenic mechanisms due to the complex network of mechanisms that drive tumor growth [230]. Conversely, models created by modifying cell lines to overexpress specific cancer biomarkers provide a clear understanding of the oncogenic mechanism. However, artificially elevating the expression of an oncogene to nonphysiological levels fails to model the intricate cascade of events that lead to tumor formation in vivo [231]. These trade-offs are significant factors contributing to the low success rates of clinical trials of targeted cancer therapies.

One way to tackle this problem is by using the growing range of CRISPR/Cas9 genome-editing tools to create tailor-made biomarker-specific cancer models from the existing library of human cancer cell lines. Emmanuelle Charpentier and Jennifer Doudna were awarded the 2020 Nobel Prize in Chemistry for their groundbreaking work in developing CRISPR/Cas9 gene editing technology, which allows precise modifications to DNA sequences in living organisms [232]. The technology consists of two main components: the Cas9 protein and a guide RNA (gRNA). The Cas9 protein is a nuclease enzyme that can cut DNA at specific locations. The gRNA is a short RNA molecule complementary to a specific DNA sequence, guiding the Cas9 protein to the target site. Delivering the Cas9 protein and gRNA to a cell makes it possible to change the DNA sequence at the target site. The mechanism of CRISPR/Cas9 involves a process that occurs naturally in certain bacteria as a defense against viral infections. Small RNA molecules called CRISPRs are a memory of past viral infections in these bacteria. When the bacteria are infected again by the same virus, the CRISPR RNA molecules guide the Cas9 protein to the viral DNA, which is then cut and destroyed [233]. The laboratory facilitates precise genetic mutations by introducing a nonfunctional version of a gene to create a knockout or loss-of-function mutation or by introducing a modified gene to create a specific point mutation. Insertion or deletion of specific DNA sequences is also possible [234]. It is possible to use CRISPR-based genome engineering to make accurate modifications to the genome of a particular cell line. This can be done to create cell lines that accurately mimic the natural development of cancer in healthy tissue or to induce specific cancer genotypes found in clinical patient samples [235]. In recent years, a number of studies utilizing CRISPR/Cas9 to create cancer cell lines for drug development and cancer biology research have emerged. The following section will delve into a few significant studies.

### Engineered cancer cell lines in cancer research

**Fig. 13:**
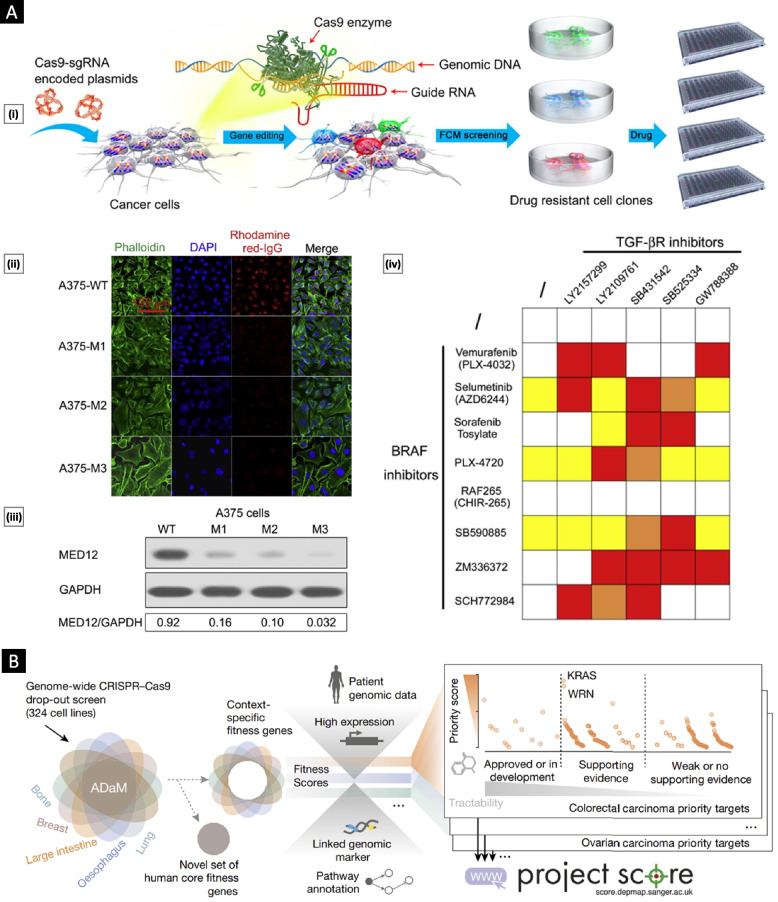
**A** Leveraging CRISPR/Cas9 to create drug-resistant cancer cell lines for use as advanced drug screening tools. Here, subfigure (i) illustrates the structure and working process of the CRISPR/hCas9 system. Subfigure (ii) depicts immunofluorescence analysis of MED12 protein levels in MED12KO cells using different stains. Cells were stained with Alexa 488-phalloidin, rabbit anti-MED12 antibody/rhodamine red-labeled goat anti-rabbit IgG, and DAPI. Subfigure (iii) shows western blot analysis confirming MED12 protein levels. Subfigure (iv) compares the response of BRAF inhibitor-resistant clones to various inhibitor combinations [Colors indicate different drug efficiencies: no significant inhibitory effect (white, cell coverage ≥ 80%), mild inhibitory effect (yellow, 60% ≤ cell coverage < 80%), moderate effect (orange, 30% ≤ cell coverage < 60%), and strong inhibitory effect (red, cell coverage < 30%)]. Adapted with permission from [236] (Copyright Elsevier, 2018). **B** A schematic overview of a novel strategy to prioritize targets in multiple cancer types by incorporating CRISPR–Cas9 gene fitness effects, genomic biomarkers and target tractability for drug development. Adapted with permission from [237] (Copyright Springer Nature, 2019)

Zhang et al*.* [236] used the CRISPR/Cas9 system to specifically knock out the Mediator complex subunit 12 (MED12) gene to develop cancer cell line-based inheritable drug-resistant models. MED12 encodes a mediator complex subunit that can confer transcriptional resistance to chemotherapy. The authors created a MED12^KO^ A375 cell model (melanoma) resistant to B-Raf proto-oncogene, serine/threonine kinase (BRAF). They reported that this cell model could be established in three weeks, much faster than the traditional method (gradient-dosage induction), which takes several months. Additionally, the induced mutations were genomically stable during passaging. A small-scale drug screening study was performed to find new combinations of drugs that could effectively treat multidrug-resistant melanoma. They focused on MED12, which blocks the glycosylation of immature forms of the TGF-β receptor, a protein that activates TGF-β signaling. Loss of MED12 leads to activation of TGF-βR signaling, which confers resistance to BRAF inhibitors. The authors showed that inhibiting TGF-βR signaling can restore drug sensitivity in cells that lack MED12. They also identified several new combinations of TGF-β inhibitors and BRAF inhibitors that showed strong synergy in suppressing drug resistance. The A375 cell line, normally sensitive to BRAF inhibitors such as vemurafenib, became resistant to these drugs by activating the TGF-βR signaling pathway. Nevertheless, the bioengineered cells regained drug sensitivity through TGF-βR inhibition (Fig. 13A).

Gonçalves et al*.* [238] employed a multifaceted approach that involved analyzing a vast dataset comprising over 199,000 drug sensitivity measurements for 397 anticancer drugs across 484 cancer cell lines. These data were integrated with genome-wide CRISPR loss-of-function screens to assess gene fitness. The study yielded valuable insights into drug targets, specificity, isoform selectivity, and drug potency. Researchers have identified robust pharmacogenomic biomarkers by scrutinizing the correlation between drug response and gene fitness data. These biomarkers hold the potential for predicting drug responses and shed light on alternative targets that could be leveraged in combination therapies. However, it is worth noting that nearly half of the drugs tested did not exhibit a significant association with gene fitness effects. Several factors could contribute to this outcome, including drug polypharmacology, distinctions between protein inhibition and knockout, incomplete target inhibition, functional redundancy among protein isoforms, and inherent limitations of CRISPR‒Cas9 screens. The authors proposed that this approach could be integrated into drug development, particularly during the hit-to-lead or lead optimization stages. Moreover, they suggested that combining this approach with other experimental and computational methods could be instrumental in investigating the mechanisms of action for novel and uncharacterized compounds. As additional data from CRISPR knockout screening and CRISPR activation and inhibition studies become available, this approach is expected to become even more valuable in unraveling cellular drug mechanisms and enhancing drug development processes.

Similarly, Behan et al*.* [237] conducted a study to identify key genes that are selectively required for the fitness of cancer cells, which could be exploited as therapeutic targets. They used CRISPR/Cas9 to disrupt genes in over 300 cancer models from 30 cancer types across 19 different cancerous tissues. By combining this information with patient genomic data, the researchers created a data-driven framework that generated a ranked list of potential new targets for various cancer types. These critical genes represent vulnerabilities in cancer cells and could contribute to the initial stages of drug development by offering a more varied and effective portfolio of cancer drug targets. The principles outlined in this study have the potential to enhance the success of cancer drug development by providing a data-driven approach to identifying new targets (Fig. 13B).

In their research on cancer metastasis and EMT, Shu et al*.* [239] developed a novel cell model using CRISPR/Cas9 technology. The aim was to create a more accurate and physiologically relevant model for studying EMT in breast cancer. To achieve this, the researchers introduced a modified gene, termed ECAD-EmGFP, into MCF10A breast epithelial cells. This modified gene allowed them to monitor the progression of EMT in real time. The key modification involved tagging EmGFP at the ECAD gene's C-terminus, a critical EMT marker. The successful knock-in of the ECAD-EmGFP gene was confirmed at multiple levels, including DNA, mRNA, and protein. Subsequently, when these engineered cells were treated with TGF-β, a well-known inducer of EMT, they underwent the EMT process. This was evident through a decrease in ECAD-GFP expression and a concurrent increase in vimentin and fibronectin expression, characteristic changes associated with EMT. Additionally, the researchers observed that the cells undergoing EMT displayed enhanced migration capabilities, a hallmark feature of EMT in cancer cells. This physiologically relevant cell model provides insights into the biology of EMT in cancer and holds promise for drug discovery efforts targeting EMT-related processes in cancer progression and metastasis.

Table 3 summarizes several studies utilizing CRISPR to generate bioengineered cancer cell lines and their application in cancer research.

**Table 3:** Bioengineered cancer cell lines using CRISPR technology

Cancer Cell Line Type	CRISPR Target Gene and Function	Key Findings/Potential Therapeutic Application	Ref
Murine melanoma B16F10 cell line	• CD47: It acts as a signal on tumor cells to prevent their phagocytosis by macrophages • IL-12: Promotes immune response against tumors by inhibiting angiogenesis, boosting T-cell growth, and inducing TAM polarization	• Tumor cells were genetically engineered to produce IL-12 while simultaneously knocking out the CD47 gene • This dual modification enhanced the immune response by promoting increased macrophage activity	[240]
Human melanoma cell lines WM115, CM150-Post, and NZM40	• Early B-cell factor 3 (EBF-3): It acts as a putative epigenetic driver of melanoma metastasis	• DNA methylation editing was conducted, with particular emphasis on the EBF-3 promoter region • Study highlights the promising capabilities of CRISPR for conducting epigenetic editing in disease-related investigations	[241]
The human NSCLC cell line A549 and the cisplatin-resistant A549 cell line (A549/DDP)	• NNT: It encodes for an enzyme known as nicotinamide nucleotide transhydrogenase, which plays a crucial role in mitochondrial energy metabolism	• CRISPR-based DNA hypermethylation leads to the silencing of NNT in cisplatin-resistant lung cancer cells • Restoring NNT expression reduces resistance to cisplatin and suppresses autophagy	[242]
ID8 cells (murine ovarian surface epithelial cells)	• Trp53/p53: A crucial tumor suppressor gene that is vital in controlling the cell cycle • Breast cancer susceptibility gene 2: Functions as a tumor suppressor gene, aiding in the prevention of cancer cell formation	• Novel ID8 derivatives were generated by introducing single or double deletions of suppressor genes • This approach enabled the modeling of high-grade serous carcinoma for further study	[243]
NPC (Nasopharyngeal carcinoma) cell line CNE2	• KLHDC4: It interacts with tumor suppressor protein p53, thereby augmenting its capacity to initiate apoptosis in cancer cells	• Gene editing of KLHDC4 led to the inhibition of cell proliferation, reduced colony formation, and suppressed tumor growth in mice • Findings suggest KLHDC4 holds promise as a potential therapeutic target and a prognostic biomarker	[244]
SH-Y5Y neuroblastoma cell line	• Chromosome 6q region: Loss of 6q genetic material, including tumor suppressor CDKN1A, affects cell growth and proliferation and is implicated in neuroblastoma progression • Chromosome 11q region: 11q genetic material loss, including tumor suppressors ATM and CBL, is linked to aggressive neuroblastoma	• CRISPR‒Cas9 mediated deletions in chromosome 11q and 6q revealed oncogenic advantages, suggesting therapeutic potential in targeting these deletions	[245]
Human cell lines Panc-1 and SUIT-2 and the murine cell line TB32047	• KrasG12D: It is postulated to be a key driver in tumor initiation and progression	• Three Kras heterozygous cell lines were investigated, and the resulting knockout clones displayed characteristics akin to those of wild-type cells during standard growth conditions	[246]
Adriamycin-resistant (A2780/ADR) ovarian cancer cell line	• ABCB1: It encodes P-glycoprotein (a membrane protein that aids in the transport of molecules across cell membranes). ABCB1 mutations are associated with anticancer drug resistance	• Downregulation of the ABCB1 results in heightened sensitivity of drug-resistant ovarian cancer cells to doxorubicin treatment	[247]
PC-9 (lung adenocarcinoma cell line)	• Epidermal growth factor receptor (EGFR): It encodes a protein that governs cell growth, survival, and migration. Abnormalities in this gene can drive cancer growth	• CRISPR/Cas9 introduced EGFR T790M mutation into PC9 lung cancer cells, creating clones resistant to gefitinib but sensitive to AZD9291	[248]

### Need for tumor organoids

Despite their enormous contributions to accelerating research, cancer cell lines are held back by their inability to replicate the intricate biology and pathophysiology of their original tumors. As an alternative, tumor organoids offer a superior and independent in vitro means of studying cancer, providing a more personalized and accurate model for studying cancer biology and testing new cancer therapies [249]. Tumor organoids are a groundbreaking development in cancer research that provides a more personalized and accurate model for studying cancer biology and testing new cancer therapies. They are 3D structures developed by embedding cancer cells in a gel-like substance that mimics the TME. They are useful in cancer research because they provide a more realistic representation of the TME than traditional 2D cell cultures, which lack the complexity and heterogeneity of real tumors [250]. They allow for the incorporation of other cell types that are present in the TME, such as immune cells, fibroblasts, and ECs. These cell types play important roles in tumor growth and response to therapy, and their inclusion in tumor organoids allows for a more realistic representation of the TME [251]. These organoids can be derived directly from patient biopsies and offer a unique opportunity to study the effects of different drugs on individual patients based on their genetic and molecular characteristics. This is particularly important for testing the effectiveness of targeted therapies, which rely on identifying specific genetic mutations or biomarkers present in the tumor [252–254].

**Fig. 14:**
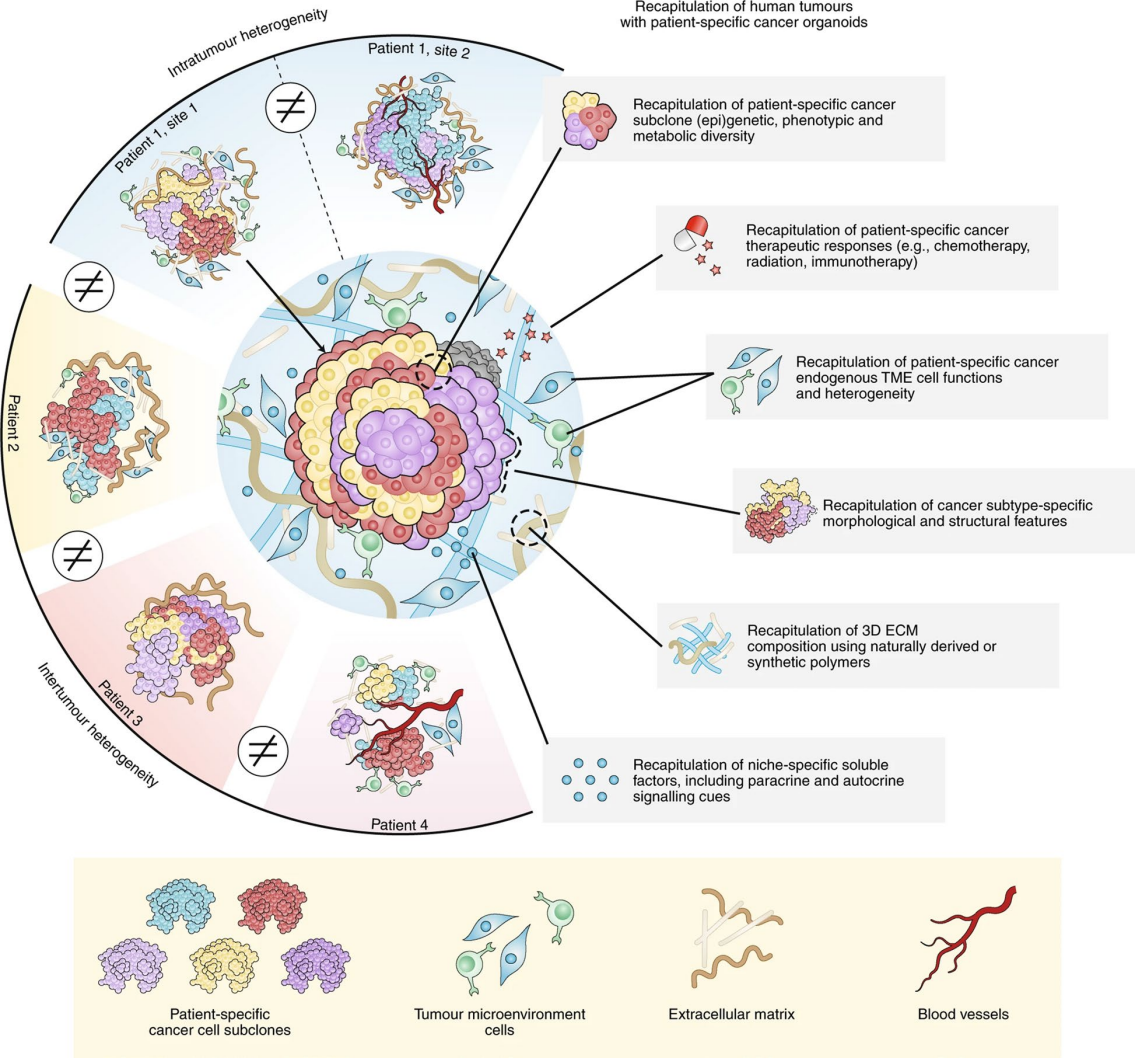
Patient-specific tumor heterogeneity reproduction in tumor organoids. Tumor organoids accurately mimic the unique characteristics of individual patients' tumors, reflecting the diverse cellular and environmental factors that contribute to tumor heterogeneity. These organoid models, derived directly from patients, effectively reproduce the phenotypic, epigenetic, and spatial diversity observed within and between tumors. Moreover, tumor organoids provide a means to study the heterogeneous TME, including the presence and functions of noncancerous TME cells, signaling through specific factors within their respective niches, and the altered composition of the ECM. Consequently, tumor organoids hold significant promise for modeling personalized responses to anticancer treatments in clinical settings. Adapted with permission from [250], (Copyright Springer Nature, 2022)

The mechanisms of cancer therapy resistance can be investigated using tumor organoids. By growing organoids from tumors resistant to a particular therapy, researchers can identify the genetic and molecular changes that lead to resistance and develop new strategies to overcome them [255]. Recent research using advanced tumor organoids and engineering approaches has enhanced our understanding of the role of immune cells in the TME. This can help researchers understand how immune cells contribute to tumor growth and identify new targets for immunotherapy [256]. Tumor organoids offer a valuable platform for investigating the impact of various microenvironmental factors on tumor development and therapeutic outcomes. For example, researchers can study the effects of hypoxia or acidosis (high acidity) on tumor growth and identify new targets for therapy [257]. By sourcing cancer cells from patients, tumor organoids can also serve as powerful models for studying tumor heterogeneity. Patient-specific tumor organoids accurately replicate the diverse genetic, phenotypic, and spatial heterogeneity of tumors [258]. These 3D models capture the genetic and epigenetic diversity of distinct neoplastic subclones and enable studying their responses to treatments. Additionally, organoids incorporate nonneoplastic TME cells, allowing the investigation of complex cellular interactions and niche-specific signaling [259]. By faithfully preserving tumor heterogeneity, tumor organoids provide a valuable tool for personalized treatment strategies and a deeper understanding of tumor biology (Fig. 14).

Overall, the reconstruction of the TME using engineered tumor organoids has enabled the discovery of novel targets and the development of more effective therapies to improve patient response rates. The following section highlights compelling instances that illustrate the distinctive applications of tumor organoids in cancer research, providing valuable insights into their utility.

### Organoids in cancer research

Dominijanni et al*.* [260] designed a 3D organoid model for investigating the intricate interactions within the TME during liver metastasis of CRC. This model was engineered to emulate the transformation of hepatic stellate cells (HSCs) into myofibroblasts, a phenomenon linked to tumor growth and resistance to chemotherapy due to excessive ECM production. The model's core consists of a collagen-based 3D hydrogel enveloping a CRC spheroid. It offers precise control over the structural and cellular aspects of the TME. Following exposure to TGF-β, a cytokine known to induce HSC activation, the TME exhibited increased stiffness attributed to heightened ECM synthesis and bundling orchestrated by LX-2 cells, akin to the behavior of CAFs in the liver. This enhanced rigidity in the TME was correlated with chemoresistance. Moreover, the organoid model revealed that collagen fibers within the TME, when influenced by TGF-β, displayed enhanced alignment, length, and width characteristics indicative of HSC activation and fibrotic tissue formation. Immunohistochemical staining revealed that cancer cells embedded within a denser ECM began expressing epithelial markers. These findings underscore the critical role of the TME in influencing cancer progression and chemoresistance. The 3D organoid model serves as a valuable tool for gaining deeper insights into TME-mediated effects on cancer cells.

Tsai et al*.* [261] addressed the need for comprehensive in vitro models of pancreatic adenocarcinoma microenvironments, which are vital for studying stromal and immune interactions within pancreatic tumors. They developed patient-matched, organotypic models encompassing human pancreatic cancer organoids, CAFs, and T cells. Their research unveiled the significant influence of fibroblasts, which secrete paracrine cytokines such as IL-6, on tumor survival and growth. These intricate in vitro models are valuable for investigating the stromal and immune components of the TME and hold promise for personalized drug testing, as organoids can be cultured from fine-needle aspiration biopsy specimens. The models offer clinical applications such as evaluating genetic and phenotypic markers, guiding individualized therapies, and aiding target identification. Moreover, they facilitate the study of immunotherapeutic strategies and immune checkpoint inhibition by incorporating lymphocytes into pancreatic cancer organotypic cultures.

**Fig. 15:**
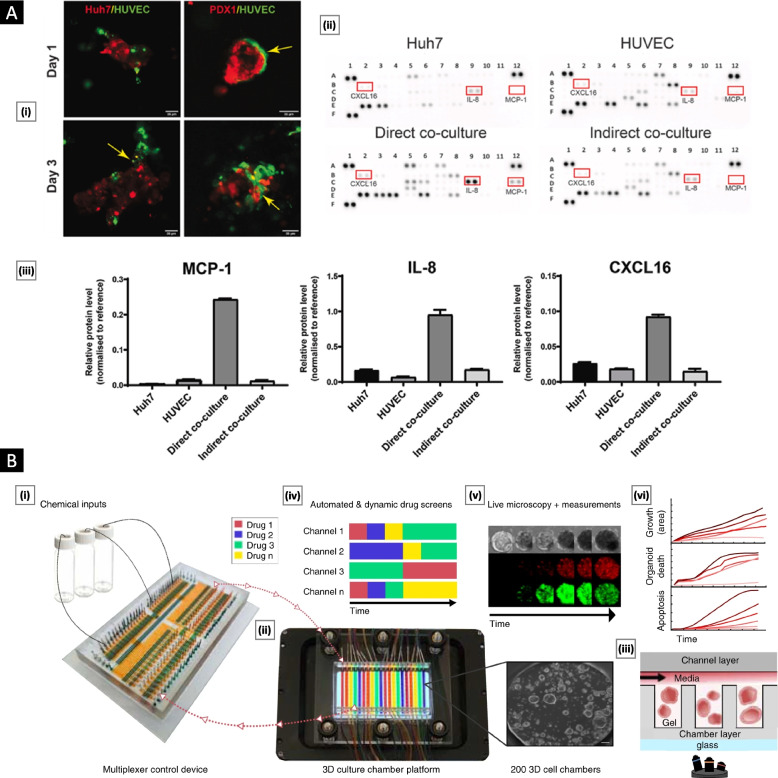
**A** Hepatocellular carcinoma organoid cocultures to understand the interplay between angiogenesis and the immune milieu. Here, subfigure (i) shows confocal microscopy images of live cocultures comprising fluorescently labeled HCC cell line-derived spheroids (Huh7) or PDX-derived organoids and ECs (HUVECs). Subfigure (ii) depicts the assessment of angiocrine signaling using the “Proteome Profiler Human Angiogenesis” antibody array. Subfigure (iii) shows the upregulation in protein levels of angiocrine factors (MCP-1, IL-8, and CXCL16) in HCC cells directly cocultured with ECs. Adapted with permission from [262] (Copyright Elsevier, 2022). **B** Automated microfluidic 3D cellular/organoid culture platform for dynamic and combinatorial drug screening. Here, subfigure (i) shows a programmable membrane-valve-based microfluidic chip that can provide automated stimulation profiles. Subfigure (ii) shows a 3D culture platform (which can be controlled by the microfluidic chip) that can produce many parallel/dynamical culture experiments. Subfigure (iii) shows a cross-section of the two-layer multichambered 3D culture chamber device (containing 200 individual chambers that are compatible with Matrigel). Subfigure (iv) depicts chemical inputs that can be preprogrammed to provide combinatorial and time-varying stimulations to the 3D culture chamber device. Subfigures (v) and (vi) show organoids or 3D cellular structures that can be continuously observed through time-lapse imaging and representative images of quantitative cellular assays, respectively. Adapted with permission from [263] (Copyright Springer Nature, 2020)

In a similar study, Lim et al*.* [262] constructed an in vitro coculture model using hyaluronic acid hydrogels to explore angiocrine interactions between ECs and hepatocellular carcinoma (HCC). This model provided insights into the potential roles of ECs in driving tumor progression independently of perfusion. The coculture induced the upregulation of MCP-1, IL-8, and CXCL16, aligning with known angiocrine signaling patterns. RNA sequencing analysis highlighted the activation of tumor necrosis factor signaling, indicating that ECs stimulate HCC cells to establish an inflammatory microenvironment that recruits immune cells. Furthermore, the model demonstrated that angiocrine crosstalk influenced macrophage polarization toward a proinflammatory and proangiogenic phenotype, resembling tumor-associated macrophages observed in HCC. This platform serves as a valuable tool for exploring the intricate interplay between angiogenesis and the immune microenvironment and assessing the clinical potential of antiangiogenic therapy in HCC (Fig. 15A).

Tumor organoids offer the potential for revolutionary high-throughput drug testing, using automation and robotics to assess multiple drugs simultaneously, expediting drug development [264]. On this note, Phan et al*.* [265] reported a high-throughput tumor organoid drug screening platform for personalized medicine. They introduced a mini-ring approach that simplifies the geometry for seeding cells around the well rims, enabling compatibility with automation and high-throughput screening. This method was tested with four patient-derived tumor organoids from ovarian and peritoneal carcinomas. They exposed these organoids to 240 kinase inhibitors and assessed viability, number, and size changes, identifying personalized responses tailored to each tumor. Impressively, the results were available within a week of surgery, making this approach rapid and efficient for informing treatment decisions. This method offers notable advantages, such as using a small number of cells, negating the need for extensive in vitro or in vivo expansion that can lead to divergence from the tumor's characteristics. It is particularly advantageous for samples that struggle to grow in vivo, reducing the time and cost of generating patient-derived xenografts (PDXs). The authors identified several effective molecules for treating a rare ovarian carcinosarcoma, which lacks a standard, optimized first-line drug regimen. This emphasizes the promise of this platform, highlighting its speed, versatility across various systems and drug screening protocols, potential for full automation, adaptability to different support materials (beyond Matrigel), and scalability to 384-well plates.

In a related investigation, Schuster et al*.* [263] developed a microfluidic 3D organoid culture and analysis system that can provide hundreds of organoid cultures with combinatorial and dynamic drug treatments, enabling real-time organoid analysis. Platform validation encompassed individual, combinatorial, and sequential drug screens on human-derived pancreatic tumor organoids. The findings demonstrated that temporally modified drug treatments exhibited superior efficacy in vitro compared to constant-dose monotherapy or combination therapy. This platform holds significant potential for advancing organoid models, enabling screening approaches closely mimicking real patient treatment courses. Consequently, it can contribute to personalized therapy decision-making. The microfluidic platform utilized in this study exhibited high reproducibility and robustness, making it well suited for accommodating complex treatment combinations and temporal sequences of culture conditions. The microfluidic architecture offers precise addition of reagents at specific times, effectively eliminating the major errors that may arise from manual approaches. Furthermore, the platform incorporates numerous repeated conditions and controls through identical well units exposed to identical conditions, enhancing accuracy. One notable advantage of the platform lies in its temporal capabilities, which facilitate testing thousands of drug combinations to replicate real-life patient treatments in a procedural manner. This feature provides valuable insights into the efficacy and potential synergistic effects of various drug combinations, aiding in identifying optimal treatment strategies (Fig. 15B).

Alternately, Maloney et al*.* [266] developed a new technique for generating high-throughput tumor organoids using an immersion printing method that employs extrusion-based bioprinting. Tumor cells were mixed with hyaluronic acid and collagen hydrogels and printed into a viscous gelatin bath. The bath provided the necessary structural support to form spheroids in 96-well plates. The study demonstrated that this technique can fabricate tumor organoids from glioblastoma and sarcoma patient biopsies and tumor cell lines, making it a promising method for generating organoids for drug screening purposes.

### Organoids in immunotherapy

Recent research has made significant advances in our understanding of the role of immune cells in the tumor TME using advanced tumor organoids and engineering approaches. The TME can impede the immune system's ability to effectively eradicate tumor cells, but immunotherapy has successfully reversed this effect. However, the variability in response among patients indicates that a deeper understanding of the mechanical interactions between immune and tumor cells is required to improve response rates and develop novel therapeutics. Immunotherapy has revolutionized cancer treatment with two key approaches: adoptive T-cell therapies and immune checkpoint blockers (ICBs). ACT introduces activated T cells to specifically target tumor cells, while ICBs boost the immune system's response by blocking inhibitory signals used by cancer cells to evade the immune system. These approaches have shown great promise in improving patient outcomes [267]. In a study by Michie et al*.* [268], the efficacy of tumor-specific cytotoxicity of T cells, specifically CAR T cells and TCR T cells, was evaluated using patient-derived organoids (PDOs) as a platform. This study investigated the impact of combining CAR T cells with birinapant, an inhibitor of apoptosis, on the growth of PDOs. The findings demonstrated that the combination therapy significantly reduced the growth of PDOs in a TNF-dependent manner, whereas CAR T cells alone showed limited efficacy. These results highlight the potential of PDOs as a valuable tool for evaluating the efficacy of combination therapies involving T cells and other targeted agents. This approach could ultimately aid in the development of more effective treatment strategies for cancer patients.

In another relevant study, Schnalzger et al*.* [221] developed a preclinical model using 3D PDOs to assess the cytotoxicity of chimeric antigen receptor (CAR) in a native tumor immune microenvironment mimicking model. They also established a live-cell imaging protocol to monitor cytotoxic activity at the single organoid level. The study demonstrated a stable effector-target cell interaction in the coculture of natural killer cells with CRC or normal organoids on an ECM layer. The authors also used CRC organoids to evaluate the tumor antigen-specific cytotoxicity of CAR-engineered NK-92 cells targeting EGFRvIII or FRIZZLED receptors. This platform can be used to assess CAR efficacy and tumor specificity in a personalized manner. Epithelial-only PDOs, which do not contain stromal or immune elements, can be used to select T cells that react to tumors. This coculture approach can be utilized to concentrate, activate, and determine the effectiveness of tumor-reactive lymphocytes [269].

Neal et al*.* [270] developed ALI (air–liquid interface) organoids by expanding and serially passaging physically processed cancer fragments using WENR (WNT3A, EGF, NOGGIN, and RSPO1) base medium. The authors demonstrated that these ALI PDOs retained stromal and immune cells and successfully reproduced the expansion, activation, and tumor cytotoxicity of TILs responding to PD-1/PD-L1 ICB, similar to the microfluidic approach. Notably, CD8 + TIL expansion, activation, and tumor cell killing were observed after just one week of anti-PD-1 treatment in ALI PDOs derived from various human tumor biopsies, including RCC, NSCLC, and melanoma. It is essential to consider the material and composition of the organoid culture devices used in immunotherapy studies, including those involving ICB. The results suggest that ALI PDOs could be an effective platform for assessing the efficacy of ICB treatments for different types of cancer.

In a study conducted by Jenkins et al*.* [271], the authors demonstrated the utility of ex vivo systems incorporating features of the TME for investigating the response to ICBs. They employed murine-derived and patient-derived organotypic tumor spheroids (MDOTS/PDOTS), which retained autologous lymphoid and myeloid cell populations and exhibited short-term responsiveness to ICB in a three-dimensional microfluidic culture. The study findings revealed that MDOTS derived from established immunocompetent mouse tumor models effectively recapitulated the response and resistance to ICB. Furthermore, the authors identified that inhibition of TBK1/IKKϵ could enhance the response to PD-1 blockade, demonstrating the predictive value of this ex vivo model in assessing tumor response in vivo. The authors propose that profiling MDOTS/PDOTS represents an innovative platform for evaluating ICB, utilizing established murine models as well as clinically relevant patient specimens. This approach holds the potential to facilitate advancements in precision immuno-oncology and the development of effective combination therapies.

## Translational considerations

**Fig. 16:**
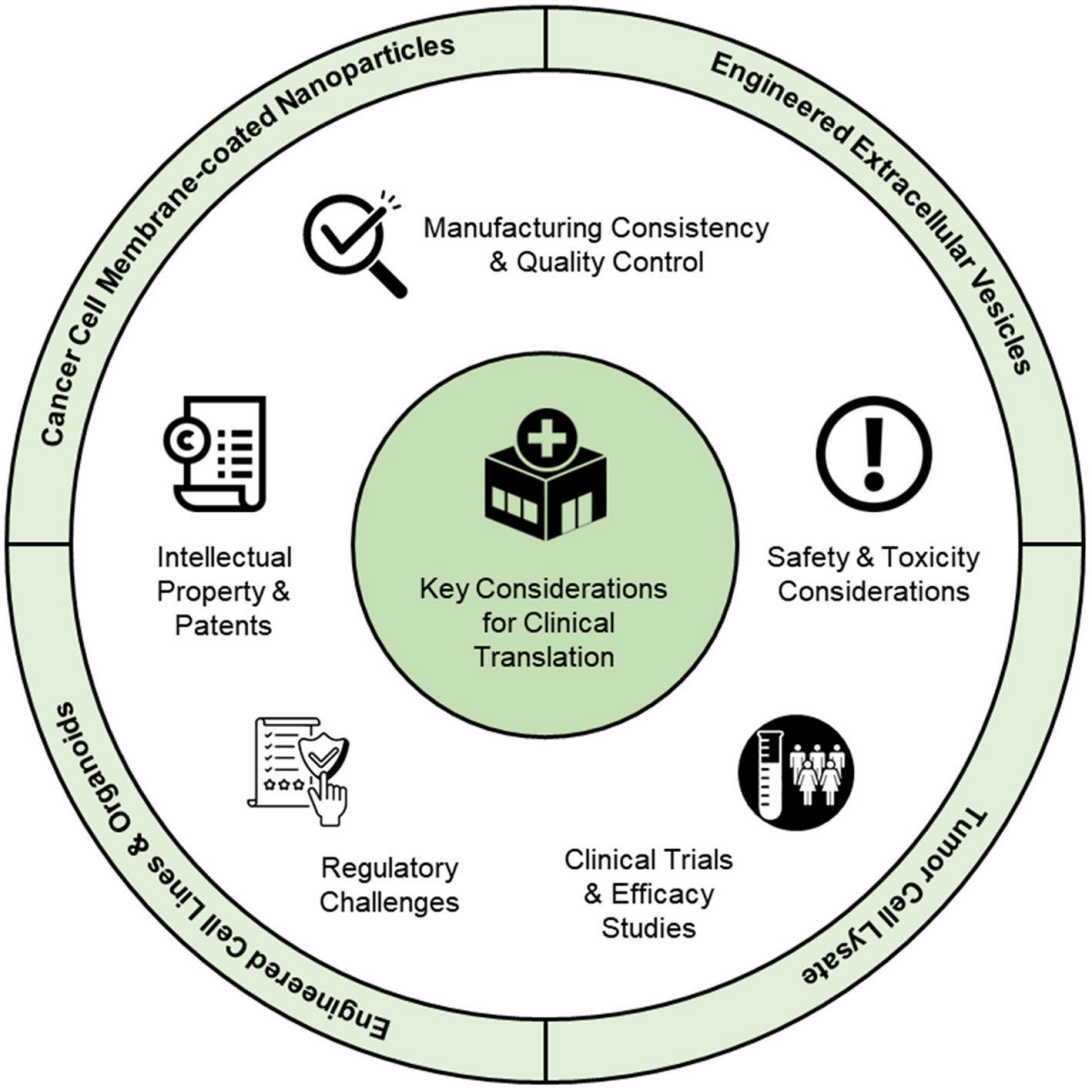
Key considerations for clinical translation of tumor-derived systems. The initial steps involve optimizing the manufacturing process and implementing stringent quality control protocols to ensure the reproducibility, scalability, and consistency of the systems. Prior to clinical translation, rigorous evaluation of safety and toxicological profiles must be conducted in preclinical stages, addressing any potential risks associated with their use. Subsequently, the efficacy of the tumor-derived systems needs to be demonstrated through well-designed clinical trials, providing robust evidence of their therapeutic potential. Obtaining regulatory approval from relevant authorities is essential for ensuring compliance with safety and efficacy standards. Additionally, addressing any intellectual property and patent-related concerns is critical to establishing ownership, encouraging innovation, and supporting commercialization efforts

Translating tumor-derived systems from laboratory research to clinical applications involves a range of complex considerations vital for their successful implementation. With their unique properties and capabilities, these systems offer exciting prospects for advancing healthcare and transforming our approach to combating cancer. By enhancing our understanding of the disease, enabling precise and early diagnosis, and facilitating personalized treatment strategies, tumor-derived systems have the potential to revolutionize cancer care. However, achieving effective translation requires careful attention to several key factors that must be considered (Fig. 16).

### Manufacturing consistency and quality control

Manufacturing consistency refers to the ability to reproduce these systems reliably and consistently across different batches and production runs. It involves establishing robust and standardized manufacturing processes that yield products with consistent properties and performance characteristics. Manufacturing consistency is important because it ensures that the tumor-derived systems used in preclinical studies accurately represent those that will be tested in clinical trials and ultimately used in patient care [272]. Achieving manufacturing consistency requires careful optimization and control of various production parameters, such as the sourcing of cancer cells/tumors, selection of raw materials, fabrication/preparation methods, manufacturing equipment, and system-specific process parameters [273]. By establishing well-defined manufacturing protocols and quality control procedures, it can be ensured that the final products meet the desired specifications consistently [274].

Quality control is another crucial aspect of manufacturing tumor-derived systems for clinical translation. It involves a set of processes and procedures designed to assess the quality, purity, and safety of the products. Quality control measures are implemented at different stages of the manufacturing process, from raw material selection to final product testing. It includes various analytical techniques and tests to evaluate the physicochemical properties, stability, and performance of tumor-derived systems [275]. These tests may involve assessing particle size and distribution, surface charge, drug loading and release characteristics, biocompatibility, and stability under various storage conditions. The use of validated analytical methods and strict adherence to quality control standards ensures that the manufactured tumor-derived systems consistently meet the required specifications [276].

Implementing robust quality control measures helps identify any manufacturing deviations or potential batch-to-batch variations that may impact the safety and efficacy of tumor-derived systems. It allows for the detection of impurities, contaminants, or any other factors that could compromise the quality of the final product. By ensuring high-quality manufacturing processes and products, manufacturers can have confidence in the reliability and reproducibility of tumor-derived systems, which is crucial for their successful translation into clinical applications [277]. Manufacturing consistency and quality control are essential not only for meeting regulatory requirements and obtaining approvals but also for building trust among clinicians, researchers, and patients. Consistent and high-quality tumor-derived systems instill confidence in the medical community and facilitate their integration into routine clinical practice. Moreover, robust manufacturing processes and quality control measures contribute to the scalability and commercial viability of these systems, making them more accessible and cost-effective for broader clinical use [278].

### Safety and toxicity considerations

Safety and toxicity considerations play a pivotal role in the successful clinical translation of any medical intervention, including tumor-derived systems. Ensuring the safety of patients is of utmost importance, and a thorough evaluation of the potential risks and adverse effects associated with these systems is critical. In the context of tumor-derived systems, safety considerations involve assessing their potential toxicity and the impact they may have on the overall health of patients. This involves examining both short-term and long-term effects, as well as evaluating the potential for systemic toxicity or damage to vital organs or tissues [279]. Preclinical studies are conducted to investigate the safety profile of tumor-derived systems before they are introduced into clinical trials. These studies involve testing the systems in laboratory models, such as animal models or in vitro cell culture models, to assess their biocompatibility and any potential harmful effects. Key parameters that are evaluated include cytotoxicity, immunogenicity, genotoxicity, and organ-specific toxicity [280].

Toxicity studies aim to identify any adverse effects caused by tumor-derived systems. This involves assessing the potential for inflammation, organ dysfunction, or other systemic responses. Various techniques and methodologies are employed to analyze the biophysical and biochemical interactions of the systems within biological systems, shedding light on their safety profile. Moreover, the potential for off-target effects must be considered. Tumor-derived systems, particularly those used for targeted therapy, should have minimal impact on healthy cells or tissues. Evaluating the selectivity of these systems and their ability to discriminate between cancerous and noncancerous cells is crucial to minimize the risk of unintended harm [281].

Safety considerations also extend to the manufacturing and storage processes of tumor-derived systems. Proper storage conditions and stability studies are conducted to ensure the integrity and efficacy of the systems throughout their shelf life. Addressing safety and toxicity concerns in the early stages of development allows researchers and clinicians to identify and mitigate potential risks. It helps refine the design and formulation of tumor-derived systems to minimize adverse effects and enhance patient safety. By comprehensively understanding the safety profile and toxicity considerations, researchers can develop robust safety guidelines and protocols for the clinical application of tumor-derived systems, thus facilitating their successful translation into clinical practice [282].

### Clinical trials and efficacy studies

Clinical trials are structured research studies conducted on human subjects to assess the safety and efficacy of new treatments, diagnostic methods, or medical devices. In the context of tumor-derived systems, clinical trials provide an opportunity to gather critical data on their performance, tolerability, and clinical outcomes. These trials are typically conducted in multiple phases, starting with small-scale studies in a limited number of patients and progressing to larger trials involving diverse patient populations [283]. Efficacy studies, on the other hand, specifically focus on evaluating the effectiveness of a particular intervention or treatment approach. In the case of tumor-derived systems, efficacy studies aim to determine the extent to which these systems can effectively diagnose, treat, or monitor cancer. These studies involve rigorous data collection and analysis to measure specific clinical endpoints, such as tumor response rates, progression-free survival, overall survival, or quality of life improvements [284].

Both clinical trials and efficacy studies are essential for establishing the safety and efficacy of tumor-derived systems in a real-world clinical setting. They provide crucial evidence that informs regulatory decisions, treatment guidelines, and clinical practice. The data generated from these studies not only support the approval and regulatory clearance of tumor-derived systems but also guide healthcare professionals in making informed decisions regarding their adoption and use [285, 286]. Moreover, clinical trials and efficacy studies help identify the patient populations that are most likely to benefit from tumor-derived systems. They contribute to the development of personalized treatment strategies by elucidating the predictive factors, biomarkers, or patient characteristics associated with positive responses to these interventions. This knowledge allows for the tailoring of treatment plans and the identification of patients who are most likely to derive significant clinical benefits [287].

Additionally, clinical trials and efficacy studies provide an opportunity to compare tumor-derived systems against existing standards of care or alternative treatment approaches. Comparative studies can demonstrate the superiority, noninferiority, or added value of tumor-derived systems in terms of safety, efficacy, or patient outcomes [288]. These head-to-head comparisons are crucial for making informed decisions about the clinical adoption and integration of these systems into routine practice [289]. The results generated from clinical trials and efficacy studies contribute to the overall body of evidence supporting the translation of tumor-derived systems into clinical practice. They provide the scientific basis for regulatory approvals, reimbursement decisions, and healthcare providers' adoption of these systems.

Additionally, these studies contribute to the ongoing refinement and optimization of tumor-derived systems, helping to improve their performance, safety profiles, and patient outcomes. Table 4 provides an overview of selected clinical trials involving tumor-derived systems. The clinical trial data presented in this table were diligently sourced from ClinicalTrials.gov (https://clinicaltrials.gov/), a reputable and comprehensive clinical trials registry. The unique NCT numbers associated with each clinical trial have been included in the table to ensure the traceability and accuracy of the referenced studies.

**Table 4:** Selected ongoing/completed clinical trials involving tumor-derived systems (Source: https://clinicaltrials.gov/)

System	NCT Number	Status	Overview of Study
*EVs*	NCT05270174	Not yet recruiting	Evaluation and validation of exosomal long noncoding RNA ELNAT1 as an independent predictor of lymph node metastasis in bladder cancer
	NCT04556916	Recruiting	Investigation of circulating blood-based biomarkers (including tumor-derived exosomes) for early detection of prostate cancer
	NCT04394572	Completed	Investigation of protein markers transported by tumor exosomes for noninvasive colorectal cancer diagnostic
	NCT02507583	Completed	The study explored immunotherapy of malignant glioma using exosomes derived from patient-derived cancer cells after treatment with an investigational antisense molecule
	NCT05286684	Recruiting	Investigation of cerebrospinal fluid microvesicles using a high-throughput clinical proteomic approach for improved profiling of metastatic tumor meningitis
	NCT01344109	Withdrawn	The study assessed the use of tumor-derived exosomes as a marker for response to therapy in women receiving neoadjuvant chemotherapy for newly diagnosed breast cancer
	NCT05397548	Recruiting	Investigation of circulating exosomal long noncoding RNA GC1 as a potential biomarker for the detection of gastric cancer
	NCT05463107	Not yet recruiting	Investigation of a potential correlation between exosomal protein biomarkers and pathological manifestation in thyroid follicular neoplasm
	NCT03334708	Recruiting	Development of a minimally invasive test for early diagnosis and treatment response monitoring in pancreatic cancer using blood-based biomarkers (including tumor-associated exosomes)
	NCT03236675	Active, not recruiting	Investigated the potential of detecting patient-specific gene rearrangement/mutation from circulating exosomes in patients of non-small cell lung cancer
*TCL*	NCT01635283	Completed	Evaluation of safety and efficacy on survival of patients with low-grade glioma, treated with autologous DCs pulsed with autologous TCL
	NCT02215837	Active, not recruiting	Evaluation of safety and efficacy of chemotherapy combined with autologous TCL-pulsed DCs for gastric cancer
	NCT00405327	Completed	Study of TCL as a vaccine for high-risk solid tumor patients following stem cell transplantation
	NCT03114631	Completed	Evaluation of safety and efficacy of novel peptide combined with TCL-pulsed DCs as immunotherapy in pancreatic cancer
	NCT01204684	Active, not recruiting	Evaluation of safety and efficacy of immunotherapy using autologous tumor lysate-pulsed DCs in patients with an intracranial brain tumor
	NCT02678741	Completed	Safety and tumor response assessment of autologous TCL, yeast cell wall particles, and DCs vaccine with checkpoint inhibitors in stage IV melanoma
	NCT01678352	Completed	Evaluation of a novel vaccination regime comprised of TCL and imiquimod (an FDA-approved immune response modifier) in glioma patients
	NCT03360708	Active, not recruiting	Evaluation of safety and feasibility of malignant glioma TCL-pulsed autologous DCs vaccine in glioblastoma patients at first or second recurrence
	NCT03395587	Recruiting	Evaluating the efficacy of integrating vaccination with TCL-loaded mature DCs into standard radio/chemotherapy for newly diagnosed glioblastoma patients
*Tumor Organoids*	NCT05842187	Recruiting	Evaluation of the consistency between in vitro tumor organoid drug sensitivity and the therapeutic efficacy of in vivo drug treatment in patients with metastatic pancreatic or gastric cancer
	NCT04777604	Not yet recruiting	Evaluation of patient-derived organoids as predictive platforms to select appropriate neoadjuvant chemotherapy before surgery, based on unique genomic mutations
	NCT05203549	Recruiting	Assessment of the consistency between treatment responses in patient-derived organoids and actual clinical outcomes in 250 gastric cancer patients
	NCT05304741	Recruiting	The study aims to establish organoid-based platforms that represent different types (advanced/recurrent/metastatic) of colorectal cancer patients and apply them to drug screening
	NCT05007379	Not yet recruiting	Development and evaluation of patient-derived breast cancer organoids to test the antitumor activity of novel chimeric antigen receptor-macrophages
	NCT04342286	Completed	Development of a reproducible organoid culture model using human kidney cells and their evaluation for developing personalized/targeted therapy
	NCT05669586	Recruiting	Evaluation of the consistency and accuracy of patient-derived lung cancer organoids, to select personalized treatment regiments for patients with resistance to multiline standard therapies
	NCT04865315	Active, not recruiting	Development of patient-derived organoids of high-grade and low-grade gliomas and their utilization in identifying underlying mechanisms that contribute to malignancy and treatment resistance
	NCT04826913	Not yet recruiting	Evaluation of a high throughput device based on 3D nanomatrices and 3D tumors with functional vascularization for personalized drug screening
	NCT05196334	Recruiting	Investigation of pancreatic ductal adenocarcinoma organoids cocultured with CAFs for pharmacotyping using relevant chemotherapeutic agents used in the clinic

### Regulatory challenges

Regulatory challenges encompass the complex regulatory framework and requirements set by regulatory agencies to ensure the safety, efficacy, and quality of medical products before they can be introduced into clinical practice. One of the primary regulatory challenges is obtaining clearance for the use of tumor-derived systems in clinical settings. Regulatory agencies, such as the FDA in the United States, require extensive preclinical and clinical data to support the safety and effectiveness of new medical technologies. This process involves rigorous evaluation of the scientific evidence, including data from in vitro studies, animal models, and clinical trials [290, 291]. Meeting these regulatory requirements can be time-consuming, costly, and challenging, requiring substantial resources and expertise. Additionally, challenges arise from the need to comply with various regulations and guidelines specific to different regions or countries. Different regulatory frameworks may exist in different jurisdictions, each with its own specific requirements and approval processes [292]. Companies and researchers must navigate these diverse regulatory landscapes to ensure compliance and obtain the necessary approvals to advance their tumor-derived systems toward clinical translation.

Another significant regulatory challenge is the evolving nature of regulatory guidelines for novel technologies. As tumor-derived systems represent innovative approaches, they may not fit neatly into existing regulatory frameworks. This can lead to uncertainties and ambiguities in determining the appropriate regulatory pathway for these systems [293]. It is crucial to engage in proactive communication with regulatory agencies to seek guidance and clarification on the regulatory requirements specific to tumor-derived systems. Collaboration between researchers, industry stakeholders, and regulatory authorities is essential to address these challenges and establish clear regulatory pathways for the clinical translation of tumor-derived systems [294]. Moreover, ensuring postmarket surveillance and monitoring is another important regulatory challenge. Once tumor-derived systems are approved for clinical use, ongoing monitoring of their safety and efficacy is necessary to identify any potential adverse effects or long-term risks. Postmarket surveillance involves collecting and analyzing real-world data from patients and healthcare providers to assess the performance and safety profile of these systems. Compliance with postmarket surveillance requirements is crucial to maintain regulatory approval and ensure the continued safe and effective use of tumor-derived systems in clinical practice [295].

### Intellectual property and patents

Intellectual property (IP) and patents play a significant role in the clinical translation of innovative technologies, including tumor-derived systems. These factors are crucial considerations due to their potential impact on the successful commercialization and adoption of these systems in clinical practice. One of the primary concerns related to IP is the need for researchers and developers to protect their novel inventions and discoveries. Obtaining patents provides legal protection and exclusive rights over intellectual property, preventing others from using, manufacturing, or selling the same technology without permission [296]. By securing patents for tumor-derived systems, researchers and companies can establish ownership and control over their innovations, which is essential for attracting investment, establishing partnerships, and commercializing the technology.

The presence of intellectual property protection fosters a competitive environment by incentivizing innovation and research and development activities. Companies and investors are more willing to invest resources and capital into translating tumor-derived systems into clinical applications when they have a strong IP portfolio [297]. The existence of patents can provide a competitive advantage, enabling the development of market exclusivity and generating revenue through licensing agreements or product sales. In the context of clinical translation, IPs and patents also contribute to technology transfer and collaboration between academia and industry. Researchers and academic institutions may collaborate with industry partners to further develop and commercialize tumor-derived systems. IP rights and licensing agreements facilitate the transfer of technology from academic settings to the private sector, enabling the necessary resources, expertise, and infrastructure for clinical translation [298].

Moreover, IP considerations also influence regulatory pathways and market entry strategies. Patents can impact the ability of other companies or organizations to develop similar technologies, creating barriers to entry for potential competitors. This exclusivity can offer a certain level of market protection and enable the patent holder to gain a foothold in the market [299]. However, it is crucial to balance IP protection with considerations of accessibility and affordability to ensure that innovative technologies, such as tumor-derived systems, are accessible to patients in need. Additionally, IP and patents can influence pricing and reimbursement considerations. The existence of patent protection can allow companies to establish pricing strategies that recoup investment costs and drive profit. However, it is important to strike a balance between recouping investment and ensuring affordability for patients and healthcare systems [300]. The cost-effectiveness and potential health benefits of tumor-derived systems must be carefully evaluated to determine their value proposition in relation to existing treatment options.

## Conclusion and future outlook

The growing body of scientific literature on tumor-derived systems underscores their pivotal role in cancer research and therapy. This suggests that this field will remain a focal point of research in the years ahead. Tumor-derived systems hold immense promise due to their inherent biological relevance, making them valuable tools for unraveling cancer biology and forging new therapeutic avenues. In this comprehensive review, we aim to explore tumor-derived systems in depth, tracing their scientific roots as potential biomedical instruments for cutting-edge applications. By delving into these systems' technical intricacies and scientific rationale, we aim to inspire the research community to propel these platforms to the forefront of our battle against cancer.

Among the array of systems discussed earlier, cancer cell-derived EVs have already made substantial strides in clinical diagnostics. EVs serve as a noninvasive source of cancer-specific biomarkers, enabling early detection, diagnosis, and disease progression monitoring. Analyzing EVs allows for the characterization of tumor heterogeneity, with different subsets of cancer EVs potentially bearing unique molecular signatures reflective of distinct tumor subpopulations. This valuable information can guide personalized treatment strategies and monitor treatment responses. Initially, EV-based cancer diagnosis faced challenges, including EV destruction due to factors such as unsuitable temperatures, tensile forces, chemicals, and prolonged storage durations [301]. Recent technological advancements have allowed the analysis of EVs using rapid yet robust techniques. Notably, commercially available and FDA-approved EV-based liquid biopsy kits, such as the ExoDx™ Lung Test (blood-based) and the ExoDx™ Prostate Test (urine-based), employ proprietary EV separation devices in conjunction with quantitative polymerase chain reaction techniques for swift diagnosis of lung and prostate cancer, respectively [302, 303]. Additionally, Exosome Diagnostics, USA, has introduced the "MedOncAlyzer 170," a pancancer liquid biopsy system that simultaneously analyzes exosomal RNA and circulating tumor DNA in a single assay to uncover functionally significant mutations across multiple cancer types [304]. While cost remains a current hurdle, several other EV-based cancer diagnosis systems, such as ExoView™ by NanoView Biosciences and ExoSearchTM by Norgen Biotek Corp., are under development and poised to reduce costs as they gain widespread usage.

While diagnostic applications of EVs are being rapidly utilized, the translation of these approaches for drug delivery has not progressed as swiftly. A similar trend is evident for cancer membrane-coated nanoparticles, which have only been applied in preclinical models for therapeutic purposes despite being conceptualized a decade ago. A potential reason for this lag is the absence of regulatory guidelines for the in vivo utilization of such systems. Given their tumor-derived origin, attributes such as pharmacokinetic profiles, long-term therapeutic safety, and toxicology require extensive study and standardization [305]. Moreover, scaling up manufacturing processes poses a significant challenge for these systems. Cancer cells are typically cultured in laboratory settings using the conventional two-dimensional flask culture method. The secreted EVs are isolated after a specific incubation period in EV-enriched media. At the same time, the CCM is collected using suitable downstream processing techniques once the cell culture flask reaches confluency.

For efficient and reproducible commercial production, it is vital to implement scalable large-scale production techniques that overcome the limitations of conventional methods. In this context, bioreactors provide controlled production environments and scalability, with the choice of bioreactor type (such as hollow fiber, membrane, or microcarrier bioreactor) and optimization of process parameters (nutrient composition, pH, temperature, dissolved oxygen levels, and agitation speed) crucial for achieving optimal productivity [306]. Optimal productivity can be achieved by selecting the appropriate bioreactor type (hollow fiber, membrane, or microcarrier bioreactor) depending on the desired output and optimizing process parameters (such as nutrient composition, pH, temperature, dissolved oxygen levels, and agitation speed). Scaling up production can also lead to reduced operating costs, lowering the final product's overall cost [307]. Both bioengineered EVs and CCM-coated nanoparticles offer unique applications, particularly those that excel in homotypic targeting, enabling them to reach challenging sites such as the bone marrow and cross the BBB [308]. By selecting the appropriate combination of nanoparticle core and delivery cargo, CCM-coated nanoparticles have been at the focal point of interesting cancer theranostic applications. With the resolution of these challenges, both tumor-derived delivery systems are poised to transition toward clinical therapeutic applications.

From an immunotherapeutic and cancer vaccine perspective, TCL has emerged as a promising tool. Researchers have demonstrated significant interest in this area, with numerous preclinical studies reporting promising results [309]. The TCL serves as a rich source of TAAs, stimulating an immune response without requiring specific antigen targeting or synthesis. This approach effectively prevents tumor evasion from immune surveillance and can be enhanced by incorporating TCL into delivery systems alongside other immunostimulatory biomolecules, generating durable immune memory to inhibit tumor relapse and metastasis. While autologous tumor cells theoretically provide an ideal source for cell lysate-based vaccines due to their unique array of tumor antigens, challenges related to limited availability and difficulty in generating large quantities of autologous tumor cells hinder widespread clinical use. In such cases, enhancing the antigenicity of allogeneic tumor cell-derived lysate remains an area for improvement [310].

Finally, engineered cell lines and tumor organoids have made significant contributions to our understanding of cancer development and drug discovery, complementing insights from traditional two-dimensional cell lines. Tumor organoids, in particular, offer distinct advantages due to their intricate cell‒cell interactions, cell–matrix interactions, and potential for cellular differentiation. These characteristics have allowed us to overcome the limitations of conventional cell lines and gain deeper insights into the complexity of cancer biology [311]. By harnessing the power of engineered cell lines and tumor organoids, we have expanded our understanding of cancer and accelerated the search for effective therapies. Tumor organoids, with their diverse cell subtypes and treatment responses more closely resembling in vivo conditions, present a promising alternative to animal-based drug testing. Increasing advocacy by global regulatory agencies for alternatives to animal testing in drug development is driven by ethical concerns and the recognition that animal models may not always accurately predict human responses. Bioengineered cell lines and tumor organoids can effectively support this effort [312].

Moving forward, stakeholders in the cell line supply industry can seize the emerging demand for organoid models by focusing on user-friendly and readily available organoid platforms. By investing in the creation of standardized organoid systems, these companies can meet the needs of researchers seeking more sophisticated and physiologically relevant in vitro models. This strategic move not only presents a lucrative business opportunity but also extends its advantages to laboratories in resource-limited countries, where establishing organoid systems from scratch can be challenging [313]. Notably, Hubrecht Organoid Technology, a nonprofit organization, is dedicated to developing a biobank of thousands of cancer-based models that closely mirror the hallmarks and diversity of human cancer. They obtain and generate organoids from patient tumor tissues, which are extensively analyzed through genome sequencing and expression profiling. The organization has created a thoroughly characterized collection of cultures along with accompanying clinical data. This resource is valuable for advancing fundamental research, identifying potential leads, and investigating innovative therapeutic approaches, offering advantages to both the industrial and academic sectors [314]. Standardized organoid assays developed through these platforms can serve as the gold standard for cancer research in the future.

Tumor-derived systems, although promising, are beset with intricate challenges. Tumors inherently manifest heterogeneity rooted in a multitude of cell types and genetic mutations. This heterogeneity, coupled with temporal biological variability within tumors, gives rise to formidable obstacles. These complexities markedly impact the precision of research outcomes in the realms of disease modeling, drug testing, and therapeutic applications. Addressing these issues necessitates rigorous standardization efforts and the development of innovative methodologies, often guided by advanced analytical techniques, to offset the influence of these inherent variations. This diligence is crucial to uphold the reliability of tumor-derived systems, ensuring their suitability for both research and clinical utility. Furthermore, the production and analysis of tumor-derived systems can be financially burdensome, curtailing their accessibility, especially within resource-constrained healthcare settings. To enhance affordability, there is an imperative need for the development of cost-effective production techniques. Collaborative endeavors, including public‒private partnerships and targeted funding initiatives, are instrumental in the pursuit of cost reduction.

While tumor-derived systems offer the potential for personalized medicine, operationalizing patient-specific treatments presents logistical complexities. Timely acquisition and processing of individual tumor samples pose practical challenges. Streamlining these procedures necessitates the integration of automation and the adoption of personalized medicine approaches to expedite the generation of patient-specific tumor-derived systems. Last, the complexity inherent in the data generated by tumor-derived systems, encompassing diverse omics data types (genomics, proteomics, etc.), poses substantial hurdles in data interpretation and analysis. Handling these intricate datasets is resource intensive and demands advanced computational methodologies. Notably, the fields of bioinformatics and machine learning hold the potential to significantly facilitate the analysis and interpretation of the multifaceted data outputs originating from tumor-derived systems, thereby enhancing their utility in cancer research and therapy.

In conclusion, tumor-derived systems, encompassing a spectrum of innovative approaches, stand as powerful allies in our ongoing battle against cancer. As we navigate the intricacies of these systems, it is evident that they offer invaluable insights into cancer biology and treatment strategies. The landscape of tumor-derived systems is poised for further exploration and development as potent biomedical tools in the fight against cancer. Their versatility, ranging from diagnostic applications such as EV-based liquid biopsies to therapeutic potentials such as membrane-coated nanoparticles, holds great promise. Overcoming challenges related to standardization, cost, patient-specific treatments, and data complexity is essential to harness the full potential of these systems. As we venture forward, it is imperative that stakeholders in the field, including researchers, clinicians, and industry partners, collaborate to address these challenges. Continued advancements in the understanding and utilization of tumor-derived systems will undoubtedly shape the future of cancer management, offering hope for improved patient outcomes and innovative approaches to cancer diagnosis and therapy.

## Data Availability

Not applicable.

## Abbreviations

APCs: Antigen-presenting cells
BBB: Blood‒brain barrier
BMDCs: Bone marrow-derived dendritic cells
BTZ: Bortezomib
CAFS: Cancer-associated fibroblasts
CCAMS: Cancer cell adhesion molecules
CCM: Cancer cell membrane
CLSM: Confocal laser scanning microscopy
CRC: Colorectal cancer
CTLs: Cytotoxic T lymphocytes
DAMPs: Damage-associated molecular patterns
DCs: Dendritic cells
ECM: Extracellular membrane
ECs: Endothelial cells
EMT: Epithelial-mesenchymal transition
EPR: Enhanced Permeability and Retention
EVs: Extracellular vesicles
FDA: Food and Drug Administration
FITC: Fluorescein isothiocyanate
gRNA: Guide RNA
H2O2: Hydrogen peroxide
HIFs: Hypoxia-inducible factors
ICBs: Immune checkpoint blockers
ICD: Immunogenic cell death
IP: Intellectual property
Ig-SF: Immunoglobulin superfamily
MDSCs: Myeloid-derived suppressor cells
MM: Multiple myeloma
MOF: Metal–organic framework
MRI: Magnetic resonance imaging
PC: Polycarbonate
PDT: Photodynamic therapy
PLGA: Poly(lactic-co-glycolic acid)
PTX: Paclitaxel
ROS: Reactive oxygen species
TAAs: Tumor-associated antigens
TAMs: Tumor-associated macrophages
TCL: Tumor cell lysate
TCVs: Therapeutic cancer vaccines
TEM: Transmission electron microscopy
TGF-β: Transforming growth factor-β
TLR: Toll-like receptor
TME: Tumor microenvironment
TSAs: Tumor-specific antigens
VEGF: Vascular endothelial growth factor
